# Pulmonary nodules: classification, biomarkers, signaling pathways, diseases, early diagnosis and management

**DOI:** 10.1038/s41392-026-03103-7

**Published:** 2026-10-13

**Authors:** Zhuomiao Ye, Lijun Li, Minghui Zhang, Wei Su, Xin Li, Shoaib Anwaar, Mingyu Xiang, Yulong Yin, Mei Liu, Mingzhu Yin

**Affiliations:** 1 Clinical Research Center (CRC), Medical Pathology Center (MPC), Cancer Early Detection and Treatment Center (CEDTC), Translational Medicine Research Center (TMRC), and CQU-Ferenc Krausz Nobel Laureate Scientific Workstation, Chongqing University Three Gorges Hospital, School of Medicine, Chongqing University, Shapingba, China; 2 Department of Medical Oncology, Harbin Medical University Cancer Hospital, Harbin, China; 3 Frazer Institute, Faculty of Health, Medicine and Behavioral Sciences, The University of Queensland, Brisbane, QLD Australia; 4 Animal Nutritional Genome and Germplasm Innovation Research Center, College of Animal Science and Technology, Hunan Agricultural University, Changsha, China; 5 Institute of Subtropical Agriculture, Chinese Academy of Sciences, Changsha, China; 6 College of Animal Science and Technology, Hunan Agricultural University, Changsha, China; 7 Yuelushan Laboratory, Changsha, China

**Keywords:** Respiratory tract diseases, Predictive markers, Translational research

## Abstract

Pulmonary nodules are increasingly detected by thoracic imaging and low-dose computed tomography-based lung cancer screening, but most are benign, transient, or indolent. Clinically, the key task is to distinguish nodules that require timely cancer-directed evaluation from those that can be safely observed. This review summarizes the classification, disease spectrum, risk assessment, early diagnosis, biomarkers, signaling mechanisms, translational models, and management of pulmonary nodules. We first distinguish imaging phenotypes and disease entities, then differentiate the individual-level models used to select candidates for lung cancer screening from the nodule-level models and structured reporting systems used after nodule detection. Low-dose computed tomography remains the foundation of screening and surveillance approaches, whereas magnetic resonance imaging (MRI), positron emission tomography combined with computed tomography, contrast-enhanced computed tomography, nonsurgical biopsy, surgical diagnosis, and local therapies play more selective roles depending on the probability of malignancy and patient fitness. Noninvasive biomarkers, including circulating cellular, nucleic acid, methylation, protein, autoantibody, extracellular vesicle, and metabolic analytes, are most likely to serve as adjuncts to imaging and clinical risk assessment rather than as stand-alone diagnostic tests. Artificial intelligence may support detection, workflow efficiency, malignancy risk reclassification, invasiveness assessment, and multimodal integration, but its value depends on external validation, calibration, implementation, and prospective evidence of management benefit. Mechanistic studies of adenocarcinoma spectrum subsolid nodules have implicated the involvement of early oncogenic signaling, genomic instability, immune escape, stromal and vascular remodeling, and adaptive survival pathways. Translational models can support mechanistic and procedural research, but do not replicate real-world nodule management in patients. Overall, care for pulmonary nodules should be risk-adapted, longitudinal, multidisciplinary, and patient-centered.

## Introduction

Pulmonary nodules are commonly found on thoracic computed tomography (CT) images, but their clinical significance varies widely, from transient inflammatory lesions to early lung cancer. The main purpose of pulmonary nodule management is to estimate malignancy risk accurately enough to guide timely diagnosis while avoiding unnecessary follow-up or invasive procedures. The prevalence of pulmonary nodules varies across regions because of differences in exposure factors and screening implementation. According to the National Lung Screening Trial (NLST) in the United States, among 26,722 participants who received low-dose CT (LDCT) screening, 24.2% of screening examinations were positive, most commonly due to the presence of noncalcified nodules.^1^ However, the NLST demonstrated that >96% of these positive cases were false positives, yielding a confirmed malignancy rate of ~3.6% among the detected abnormalities.^1^ The International Early Lung Cancer Action Program (I-ELCAP) conducted LDCT screening on 57,496 asymptomatic individuals and revealed that 17,356 (30.2%) had solid nodules (SNs), 2892 (5.0%) had mixed ground-glass nodules (mGGNs), and 2392 (4.2%) had pure ground-glass nodules (pGGNs).^2^ Notably, longitudinal follow-up in the I-ELCAP cohort revealed that only 3.1% (73/2392) of patients with pGGNs were ultimately diagnosed with lung cancer. In Japan, data from 7956 participants at health check-ups revealed a pulmonary nodule detection rate of 26.3%, yet primary lung cancer was confirmed in only 0.44% of the participants during the baseline screening.^3^ During the same period, a study from the Samsung Medical Center in Korea reported that among 6406 asymptomatic participants aged 45 and older, 35.0% were found to have pulmonary nodules, yet the overall lung cancer detection rate was only 0.36%.^4^

With the wider use of CT and LDCT lung cancer screening, pulmonary nodules are being detected more frequently. According to data from two large Dutch hospitals, the proportion of patients with new nodules increased from 9% in 2010 to 17% in 2017.^5^ Age is a critical factor in the prevalence of pulmonary nodules. In a survey of a Nordic nonsmoking population, the prevalence of nodules was 39.4% among men aged 45–49.9 years, and it increased among those aged 80 years and older.^6^ Furthermore, although most studies have shown an increase in the prevalence of nodules with increasing age, some studies have reported a U-shaped relationship between age and pulmonary nodules, suggesting that different risk factors may be present in younger and older age groups.^5^ This may be related to smoking habits, environmental exposures, and biological differences.

Widespread LDCT screening has improved the detection of pulmonary nodules, but it has also made postdetection decision-making more complex. The clinical task is not simply to find more nodules but also to identify which lesions are likely to represent clinically important early lung cancer and which can be safely observed. Imaging phenotype, detection context, growth behavior, patient-level risk, and fitness for invasive procedures all influence this judgment. Emerging tools, including liquid biopsy and artificial intelligence (AI), may refine risk assessment in certain contexts. Animal models are discussed mainly for mechanistic and procedural research, and traditional Chinese medicine (TCM) is reviewed as an area of exploration with limited current evidence for routine pulmonary nodule management.

**Fig. 1:**
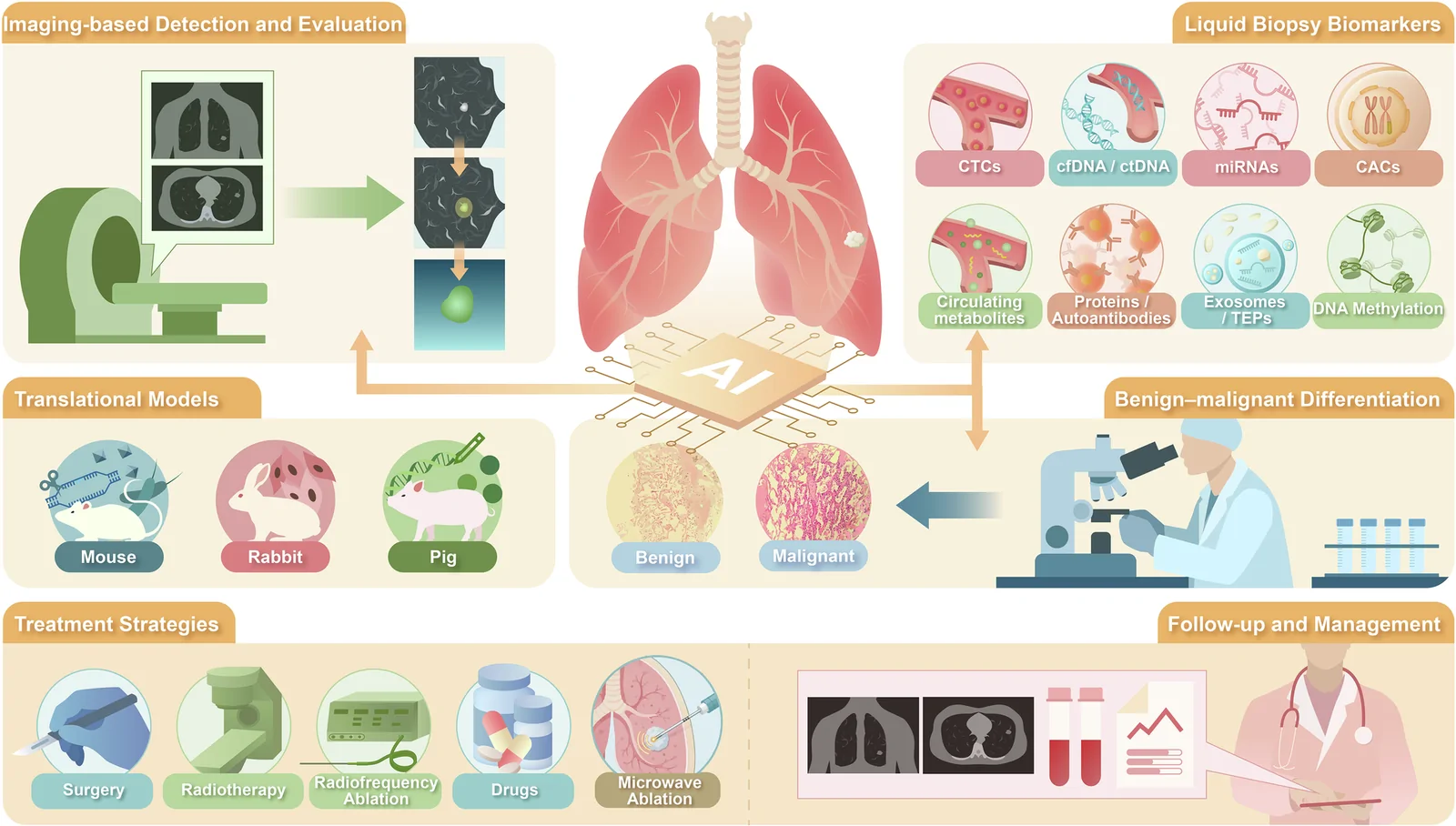
Integrated framework for pulmonary nodule evaluation and management. Schematic summarizing the principal domains involved in pulmonary nodule evaluation and management. Imaging provides the foundation for nodule detection, morphological characterization, quantitative measurement, and longitudinal assessment. Artificial intelligence may support image analysis, risk estimation, and multimodal data integration, whereas liquid biopsy biomarkers may provide complementary biological information in certain clinical or investigational contexts. Histopathologic and molecular assessments remain important when tissue diagnosis is indicated. Translational animal models support mechanistic investigations and the development and validation of imaging, localization, biopsy, and interventional techniques, but they do not fully reproduce the natural history of human pulmonary nodules. Management options range from imaging surveillance and etiology-directed treatment to surgery, radiotherapy, and image-guided ablation, based on malignancy probability, longitudinal behavior, patient fitness, and preferences. The arrows indicate conceptual relationships rather than a mandatory clinical sequence. AI artificial intelligence, CACs circulating genetically abnormal cells, cfDNA cell-free DNA, CTCs circulating tumor cells, ctDNA circulating tumor DNA, miRNAs microRNAs, TEPs tumor-educated platelets. Created using Adobe Illustrator

This review links imaging classification, disease spectrum, screening risk assessment, nodule-level malignancy estimation, diagnostic work-up, biomarker-assisted risk refinement, AI, molecular mechanisms, translational models, and guideline-based management. The aim is to provide a clinically oriented synthesis for clinicians and researchers while distinguishing established management tools from investigational approaches. An integrated framework linking pulmonary nodule evaluation, risk refinement, translational research, and clinical management is presented in Fig. 1.

## Evaluation of pulmonary nodules

### Classification by number and morphology

Pulmonary nodules are typically classified according to their number and CT morphology. Based on their number, pulmonary nodules can be classified as solitary or multiple. A solitary pulmonary nodule refers to a single focal pulmonary lesion, whereas two or more lesions are classified as multiple pulmonary nodules. This distinction is clinically relevant because multiple nodules require evaluation of each lesion, with particular attention given to the dominant or most suspicious nodule.

On the basis of morphology or composition, pulmonary nodules can be divided into solid nodules and subsolid nodules.^7–9^ Solid nodules appear as round or oval areas of increased attenuation that obscure underlying vascular and bronchial structures. Subsolid nodules include pGGNs and part-solid nodules (PSNs). pGGNs appear as areas of increased attenuation that do not obscure underlying vascular or bronchial structures and contain no solid component. The presence, size, and growth of the solid component are important indicators of invasive potential.

### Nodule distribution and quantitative descriptors

According to the Fleischner Society terminology for thoracic imaging, the location of nodules can be described as peribronchovascular or centrilobular, depending on their spatial relationship to surrounding structures.^10^ Various guideline frameworks also provide specific descriptors for airway nodules, atypical pulmonary cysts, pleural nodules, pleural-based nodules, fissural nodules, and perifissural nodules, offering more precise localization and classification criteria.^8,11,12^

Size, volume, growth rate, solid component size, and volume doubling time are not density classes but quantitative imaging descriptors that strongly influence malignancy risk stratification and management. These descriptors should therefore be interpreted together with nodule morphology, location, detection context, and longitudinal change.

### Disease spectrum and clinical implications

Pulmonary nodules are radiological findings with a broad disease spectrum rather than a single pathological entity. They may represent transient inflammatory opacities, infectious granulomas, hamartomas, intrapulmonary lymph nodes, premalignant adenocarcinoma spectrum lesions, invasive primary lung tumors, or metastases. This heterogeneity explains the need to interpret imaging findings together with clinical context, longitudinal behavior, and malignancy risk assessment.

## Risk stratification models for patients and nodules

Risk assessment for pulmonary nodule management operates at two levels. Individual-level assessment estimates the future risk of lung cancer incidence or death, is independent of any specific detected nodule, and is used to select candidates for LDCT lung cancer screening. Nodule-level assessment estimates the likelihood that an existing pulmonary nodule is malignant and guides surveillance, nonsurgical biopsy, surgical diagnosis, or treatment. These tools differ in purpose, derivation cohort, baseline disease prevalence, and validation setting. The Lung CT Screening Reporting and Data System (Lung-RADS) and Chinese Lung Imaging Reporting and Data System (C-Lung-RADS) provide additional structure for reporting and management, but they are not equivalent to multivariable prediction models. Representative LDCT screening trials, individual-level screening models, nodule-level malignancy models, and structured reporting frameworks are summarized in Table 1.

**Table 1:** Representative lung cancer screening studies, risk-prediction models, and structured reporting frameworks

Evidence domain	Study/model	Design and source population	Clinical role	Key intervention/inputs	Key message	Caveats and appropriate use
LDCT screening evidence	NLST	Multicenter RCT; 53,454 high-risk current or former smokers aged 55–74 years in the United States	Foundational mortality evidence for LDCT lung cancer screening	Three annual LDCT rounds versus chest radiography	LDCT reduced lung cancer mortality by 20% and all-cause mortality by 6.7%	High false-positive burden; comparator was chest radiography rather than usual care
LDCT screening evidence	NELSON	Population-based RCT; 15,792 high-risk participants in the Netherlands and Belgium	Mortality evidence using volume-based nodule management	Volume-based LDCT and volume-doubling time assessment versus no screening	Reduced lung cancer mortality under a volume-based nodule-management strategy, with relatively low referral rates	The female subgroup was smaller, and estimates were less precise; the trial was primarily powered around the male cohort
LDCT screening evidence	MILD	Randomized LDCT screening trial; 4099 smokers or former smokers in Italy	Evidence for prolonged and biennial LDCT strategies	Annual or biennial LDCT versus no intervention	At 10 years, LDCT screening was associated with lower lung cancer mortality, with the benefit becoming more apparent during extended follow-up	Modest sample size; earlier analyses were underpowered
LDCT screening evidence	DANTE	Randomized trial; 2450 male smokers or former smokers aged 60–74 years in Italy	Trial evidence with a non-LDCT control design	Annual LDCT versus a non-LDCT control strategy	More stage I cancers and resections were detected in the LDCT arm, but mortality was not significantly reduced	Limited statistical power; more downstream testing and invasive procedures in the LDCT arm
LDCT screening evidence	PanCan	Prospective single-arm multicenter study; 2537 ever-smokers aged 50–75 years in Canada	Model-based screening selection	Eligibility based on predicted 6-year lung cancer risk rather than fixed pack-year criteria	High cancer yield and early-stage enrichment, supporting risk-based screening selection	No randomized comparator; mortality benefit cannot be directly inferred
LDCT screening evidence	I-ELCAP	Prospective multi-institutional screening cohort; 89,404 participants internationally	Long-term observational evidence for repeated annual LDCT	Standardized annual LDCT protocol	81% clinical stage I; 10- and 20-year lung cancer-specific survival, both 81%	Cohort study rather than RCT; supports survival and stage-shift evidence rather than direct mortality reduction
LDCT screening evidence	COSMOS	Prospective single-center CT screening cohort; risk model based on 5203 asymptomatic high-risk participants in Italy	Risk-based selection for LDCT screening	Participant risk factors and lung cancer occurrence in the COSMOS screening cohort	Supported individualized risk stratification for selecting smokers for CT screening	Single-center cohort; external validation is required; no randomized mortality comparison
LDCT screening evidence	ITALUNG	Population-based RCT; 3206 high-risk individuals aged 55–69 years in Italy	Feasibility of community-based LDCT screening	Four annual LDCT rounds versus usual care	Demonstrated feasibility and early-stage detection in a population-based setting	Recruitment efficiency was limited; baseline positive findings increased downstream diagnostic assessment
LDCT screening evidence	PLuSS	Prospective single-arm cohort; 3642 current or former smokers aged 50–79 years in the United States	Benefit–harm data from an early clinical screening program	Baseline and repeat annual LDCT with 3-year follow-up	Detected more early-stage cancers but generated substantial follow-up imaging and benign procedures	Non-randomized, single-center design; no mortality comparison
Person-level screening risk models	PLCOm2012	Model developed and validated using PLCO data	Screening eligibility assessment	Age, race/ethnicity, education, BMI, COPD, personal cancer history, family history, and smoking variables	Better discrimination and sensitivity than the NLST eligibility criteria in several validation settings	Developed largely in North American populations; calibration may vary externally
Person-level screening risk models	LLP	The case-control model was later validated in UK screening cohorts	Screening eligibility assessment	Age, sex, smoking duration, prior pneumonia, asbestos exposure, family history, and prior malignancy	Provided a risk-based selection tool used in UK screening research	Performance depends on calibration to the target population
Person-level screening risk models	LCRAT/LCDRAT	Developed in PLCO ever-smokers and validated in PLCO, NLST, and external population-based datasets	Incidence and mortality risk estimation in ever-smokers	Demographics, smoking exposure, BMI, emphysema, family history, and related clinical variables	Outputs individualized 5-year lung cancer incidence risk and death risk	Transportability and calibration require validation in non-source populations
Person-level screening risk models	China NCC-LCm2021	Multicenter prospective Chinese cohorts; separate models for never-smokers and ever-smokers	Screening eligibility assessment in Chinese populations	Age, sex, BMI, smoking exposure, family history, and chronic respiratory disease	Outputs individualized 3-year lung cancer risk and improves risk enrichment in Chinese populations	Follow-up is relatively short; long-term effectiveness and cost-effectiveness need further evaluation
Person-level screening risk models	TNSF-SQ	Two-stage absolute-risk model in Asian never-smoking women, with independent validation	Risk assessment in Asian never-smoking women	Age, BMI, COPD, education, family history, and selected SNPs	Outputs 6-year risk and identifies higher-risk never-smoking women	Derived from case-control data, prospective screening calibration remains needed
Person-level screening risk models	Korean lung cancer risk model	Population-based Korean NHIS cohort model in ever-smokers	Screening eligibility assessment in Korean ever-smokers	Age, sex, smoking exposure and cessation, BMI, lifestyle factors, and pulmonary disease history	Outputs medium-term lung cancer risk and improves sensitivity/PPV over fixed criteria	Developed within a single national health-screening system, external validation remains limited
Nodule-level malignancy models	Mayo	Clinical prediction model derived from 629 patients with solitary pulmonary nodules of 4–30 mm	Pretest malignancy estimation for clinically detected nodules	Age, smoking history, history of extrathoracic cancer more than 5 years earlier, nodule diameter, upper-lobe location, and spiculation	Outputs pretest malignancy probability and remains widely used in clinical nodule assessment	Derived in clinical referral-era cohorts; calibration varies, and use in screen-detected nodules is limited
Nodule-level malignancy models	Brock	Developed and validated in baseline LDCT screening cohorts	Malignancy estimation for screen-detected nodules	Age, sex, family history, emphysema, nodule size, type, location, count, and spiculation	High discrimination and calibration in LDCT screening cohorts	Best suited to baseline screen-detected nodules in LDCT cohorts; not directly generalizable to incidental or high-prevalence referral nodules
Nodule-level malignancy models	Herder	Referral cohort of 106 patients with indeterminate solitary pulmonary nodules	PET-refined malignancy estimation	Mayo-type pretest probability plus visually graded FDG-PET uptake	Improves discrimination compared with clinical pretest models alone	Developed in a high-prevalence referral population; not a first-line screening model
Nodule-level malignancy models	TREAT 2.0	Multicenter specialist-referral cohorts; 1401 patients with high cancer prevalence	Malignancy estimation in high-risk specialty nodule evaluation	Clinical setting, demographics, smoking, symptoms, imaging findings, growth, lung function, prior cancer, and PET	Better calibration and discrimination than several older models in specialty settings	Designed for high-prevalence specialist referral practice rather than general LDCT screening
Structured reporting/management frameworks	Lung-RADS	ACR structured reporting and management system for LDCT screening	Standardized management of screen-detected nodules	Nodule type, size, growth, new or stable status, and special categories such as airway nodules, juxtapleural nodules, and atypical cysts	Standardizes LDCT screening reports and reduces false-positive classifications, with potential trade-offs depending on threshold and setting	Not a multivariable malignancy model; applies to LDCT lung cancer screening settings
Structured reporting/management frameworks	C-Lung-RADS	Data-driven Chinese CT-based risk-stratification framework	Structured risk stratification for pulmonary nodules in Chinese CT cohorts	CT-derived nodule features and risk-stratification categories	Represents a population-adapted framework for pulmonary nodule risk management in China	Further prospective implementation and external validation beyond Chinese CT cohorts are needed

LDCT screening studies, person-level screening risk models, nodule-level malignancy models, and structured reporting systems address different clinical questions. Screening trials evaluate whether LDCT-based lung cancer screening reduces mortality; person-level models select individuals for screening; nodule-level models estimate the probability of malignancy after a nodule has been detected; and structured systems standardize reporting and management. These tools should not be used interchangeably

*ACR* American College of Radiology, *AUC* area under the receiver operating characteristic curve, *BMI* body mass index, *COPD* chronic obstructive pulmonary disease, *FDG-PET* fluorodeoxyglucose positron emission tomography, *LDCT* low-dose computed tomography, *NHIS* National Health Insurance Service, *PET* positron emission tomography, *PPV* positive predictive value, *RCT* randomized controlled trial, *SNP* single-nucleotide polymorphism, *VDT* volume-doubling time

### Individual-level risk assessment and screening eligibility

Randomized trials have established LDCT as an effective lung cancer screening strategy in selected high-risk populations. Compared with chest radiography, the NLST showed reduced lung cancer mortality with annual LDCT screening, and the NELSON study confirmed the benefit of volume-based LDCT screening compared with no screening.^1,13^ These trials shaped eligibility criteria centered on age, smoking exposure, and smoking cessation history. However, simple rule-based thresholds do not fully capture individual risk and have motivated the evaluation of multivariable individual-level risk models for screening selection.^14–16^

Several landmark studies have helped define high-risk populations. NLST enrolled individuals aged 55–74 years with at least 30 pack-years of smoking exposure and, for former smokers, cessation within 15 years.^1^ NELSON enrolled individuals aged 50–74 years with heavy smoking exposure and cessation within 10 years,^13^ whereas I-ELCAP provided long-term observational evidence from a large international LDCT screening cohort.^17^ PanCan illustrated a model-based approach by enrolling participants according to predicted lung cancer risk.^18^ Beyond these landmark studies, MILD and DANTE provided complementary evidence in terms of screening intervals and longer-term outcomes, whereas ITALUNG documented the feasibility and baseline performance of a population-based randomized LDCT screening trial.^19–21^ PLuSS characterized early screening outcomes and the diagnostic work-up prompted by CT-detected abnormalities, whereas COSMOS informed the risk-based selection of smokers for LDCT screening.^22,23^ The UKLS and community-based Lung Health Check programs also contributed evidence on smoking cessation and real-world implementation.^24,25^

Guideline definitions of high-risk populations vary, but most are anchored in age, smoking exposure, and suitability for curative-intent treatment. The 2021 US Preventive Services Task Force (USPSTF) recommends annual LDCT screening for adults aged 50–80 years with a smoking history of at least 20 pack-years who currently smoke or have quit smoking within the past 15 years.^26^ The National Comprehensive Cancer Network (NCCN) recommends LDCT screening for individuals at high risk, with eligibility and termination decisions informed by individualized risk, shared decision-making, and candidacy for curative treatment.^12^ Recent NCCN recommendations use lower age and smoking exposure thresholds than earlier NLST-based criteria do, reflecting evidence from randomized trials, cohort studies, and modeling analyses.^12,27–29^ Differences between the USPSTF, Centers for Medicare & Medicaid Services (CMS), and NCCN recommendations regarding quit-years and termination criteria should be interpreted in light of the specific guideline version.^12,26,30^ Guidelines generally support continued screening while curative-intent treatment remains appropriate; longitudinal studies support ongoing risk assessment after negative or low-risk baseline findings.^12,31–33^

Because age- and smoking-based criteria incompletely capture lung cancer risk, several individual-level prediction models have been developed to refine screening selection. Representative models include PLCOm2012, the Liverpool Lung Project (LLP) model, the Lung Cancer Death Risk Assessment Tool (LCDRAT), and the Lung Cancer Risk Assessment Tool (LCRAT). PLCOm2012 incorporates demographic, smoking-related, and clinical variables and has shown better discrimination than the NLST eligibility criteria have.^16,34^ The area under the receiver operating characteristic curve (AUC) values for PLCOm2012 were 0.803 and 0.797 for the development and validation datasets, respectively, and the sensitivity and positive predictive value were higher than those of the NLST criteria without the loss of specificity.^16^ Prospective data from the International Lung Screening Trial similarly revealed that compared with the USPSTF 2013 criteria, PLCOm2012 identified more individuals who subsequently developed lung cancer.^14^ The LLP model was derived from 579 lung cancer cases and 1157 matched controls and was later refined and validated in UK screening cohorts.^35–38^ LCRAT and LCDRAT estimate the 5-year lung cancer incidence and death risk among ever-smokers and have shown strong discrimination in selected validation contexts.^28,39^ These models are designed for screening selection and should not be used to estimate the malignancy probability of a detected nodule.

The clinical value of individual-level models also depends on fairness and transportability. Eligibility based only on age and pack-years may introduce sex- and race-related disparities,^40,41^ whereas PLCOm2012-based approaches may reduce some of these biases.^42–44^ This issue is particularly relevant in East Asia, where Western-derived models may be less generalizable due to differences in smoking patterns, sex distribution, lung cancer histology, molecular profiles, environmental exposure, and underlying pulmonary disease.^44–46^ A family history further increases the risk of lung cancer among never-smokers and light smokers. A prospective LDCT screening study of 1102 asymptomatic first- and second-degree relatives of patients with lung cancer reported a detection rate of 4.5%, with a higher risk in multiplex families,^47^ and a meta-analysis confirmed the association between first-degree family history and lung cancer risk.^48^

Several Asian countries have therefore developed population-adapted risk frameworks. Korea has proposed models incorporating demographic, smoking-related, and pulmonary disease-related variables, with improved calibration and discrimination over traditional Western criteria in Korean ever-smokers.^49^ Chinese recommendations define high-risk individuals as adults aged ≥50 years with at least one major risk factor and generally recommend LDCT screening for high-risk individuals aged 50–74 years without prior lung cancer diagnosis.^50^ Dedicated models for never-smokers are also emerging. China NCC-LCm2021 developed separate 3-year risk models for never-smokers and ever-smokers from a prospective Chinese cohort and proposed screening thresholds for never-smokers.^51^ The TNSF-SQ model for never-smoking Asian women achieved moderate discrimination in an external validation cohort.^52^ At present, however, LDCT screening in never-smokers is better viewed as a risk-enriched research direction than as a universal screening strategy. Population-based studies from East Asia have raised concerns about potential overdiagnosis, showing increased detection of early-stage lung cancer without a corresponding increase in late-stage incidence.^53,54^

### Nodule-level models for malignancy probability assessment after nodule detection

Nodule-level models are used after a pulmonary nodule has been detected. Their purpose is to estimate the probability of malignancy and to guide surveillance, nonsurgical biopsy, surgical diagnosis, or treatment decision-making. Most models combine clinical and imaging features, and some incorporate metabolic imaging.

The Mayo model is a classic malignancy prediction model for radiologically indeterminate solitary pulmonary nodules.^55,56^ It was developed and validated using a cohort of 629 patients and includes age, smoking status, prior cancer history, nodule diameter, spiculation, and upper lobe location.^55^ Subsequent validation studies have shown good but variable performance across clinical contexts, particularly in contemporary cohorts with incidentally detected nodules.^56,57^ The Herder model refines the Mayo-type pretest probability by incorporating fluorodeoxyglucose positron emission tomography (FDG-PET) uptake and is most useful once an indeterminate nodule has entered further diagnostic workup.^58^ In a validation study of 106 patients with relatively high cancer prevalence, the Herder model reported an AUC of 0.88, with threshold-dependent sensitivity and specificity.^58^ Because the Mayo and Herder models were developed or validated primarily in clinically evaluated or referral-type populations, they are most applicable to incidental or diagnostically evaluated indeterminate nodules rather than unselected LDCT screening populations.

The Brock model is more closely aligned with the screening context. It was developed from 12,029 nodules in 2961 participants from the PanCan and British Columbia screening cohorts, includes 144 malignant nodules, and incorporates age, sex, emphysema status, family history, nodule size, location, type, count, and spiculation. In the validation cohort, it showed excellent discrimination, with an AUC >0.90.^59^ Because Brock was derived from screening cohorts, it is particularly suited to nodules detected by baseline screening; application to new or incident nodules during serial LDCT screening should consider the validation context.^59^

TREAT 2.0 was developed and validated in multicenter specialist referral cohorts of 1401 patients with an overall lung cancer prevalence of 70%.^60^ It includes 12 variables and updated strategies for handling missing data. For high-risk indeterminate pulmonary nodules, TREAT 2.0 achieved an AUC of 0.85 and outperformed Mayo, Herder, and Brock in this specialist referral setting.^60^ Its high-prevalence validation context, however, limits direct extrapolation to lower-risk screening contexts. Mayo, Herder, Brock, and TREAT 2.0 are all nodule-level malignancy models, but their derivation cohorts, disease prevalence, input variables, and intended use differ substantially. Model choice should therefore depend on clinical context rather than the AUC alone.

The detection context is central to model selection. Screen-detected nodules are found in organized LDCT lung cancer screening programs, usually in asymptomatic individuals, and are evaluated in a lower-prevalence setting where the main challenge is balancing overinvestigation with missed cancerous nodules. Models derived from screening cohorts, such as Brock, are better aligned with this scenario.^59,61^ A post hoc UKLS analysis revealed that, among screen-detected SNs or PSNs, a baseline volume of at least 300 mm³ and upper lobe location were associated with a higher 2-year lung cancer risk; nodules of at least 300 mm³ had a 2-year cancer probability of 22.1%, compared with 1.3% for nodules measuring 100–300 mm³.^62^ Thus, imaging features such as volume and location remain central to screening-based risk stratification alongside formal prediction models.

Incidentally detected nodules are also identified outside organized lung cancer screening programs, including opportunistic health check CT or imaging performed for other clinical indications. These populations are more heterogeneous and may include patients with prior malignancies, symptoms, chronic lung disease, or indication-related selection factors. They more closely resemble the clinical contexts in which Mayo, Herder, and TREAT 2.0 were developed or validated.^57,58,60^ Screen-detected and incidentally detected nodules should be regarded as distinct clinical contexts, and malignancy models should be matched to the detection setting to support appropriate risk estimation and management.

### Structured reporting systems and guideline-based risk stratification frameworks

Pulmonary nodule management also relies on structured reporting systems and guideline-based risk frameworks. These tools do not primarily estimate future lung cancer risk at the person level or generate a precise individualized malignancy probability for a given nodule. Instead, they standardize image interpretation, harmonize risk categories, and link findings to management recommendations. They are best viewed as reporting and management frameworks rather than formal multivariable prediction models.

Lung-RADS was introduced by the American College of Radiology to standardize the categorization and management of screen-detected nodules.^61^ It classifies findings into categories 0, 1, 2, 3, 4 A, 4B, and 4X, ranging from incomplete examination and negative findings to suspicious or very suspicious lesions.^11,61^ Lung-RADS standardizes LDCT screening reports and reduces false-positive results.^61,63^ In retrospective reclassification of NLST data, Lung-RADS reduced the baseline false-positive rate from 26.6% to 12.8%.^64^

The American College of Chest Physicians (ACCP) and the Fleischner Society guidelines provide risk frameworks that inform management decisions.^8,9^ In general, younger age, limited or no smoking exposure, smaller size, smooth margins, and non-upper lobe location indicate lower risk, whereas older age, substantial smoking exposure, larger size, irregular or spiculated margins, and upper lobe location indicate higher risk.^8,9^ The 2024 Chinese Expert Consensus on Pulmonary Nodules adopted an integrated framework based on clinical features, FDG-PET findings, nonsurgical biopsy results, and CT follow-up observations, classifying nodules as low, intermediate, or high risk.^65^ C-Lung-RADS, developed and validated on CT data from large Chinese cohorts, provides a locally adapted structured framework for pulmonary nodule risk management in the Chinese population.^66^

Pulmonary nodule management, therefore, requires distinction among three categories of tools: individual-level risk models for selecting candidates for LDCT lung cancer screening, nodule-level malignancy models for estimating the cancer probability of an already detected nodule, and structured reporting or guideline-based management frameworks. Keeping these categories separate helps align screening selection, postdetection risk estimation, and downstream management.

## Imaging and diagnostic work-up

Imaging is the first step in evaluating pulmonary nodules and remains the primary framework for subsequent diagnostic decisions. Within a CT-centered imaging framework, LDCT anchors lung cancer screening and longitudinal nodule surveillance, whereas contrast-enhanced CT (CE-CT), particularly dynamic contrast-enhanced CT (DCE-CT), may provide selective postdetection information on enhancement behavior and vasculature-related lesion characterization. MRI and positron emission tomography/computed tomography (PET/CT) provide complementary information for radiation-free follow-up, metabolic activity, staging, and selected diagnostic work-up. When a clinically important probability of malignancy remains after imaging and clinical risk assessment, tissue diagnosis may be pursued through percutaneous or bronchoscopic approaches. Preoperative localization is discussed separately because it facilitates minimally invasive resection of small or impalpable nodules but does not establish a pathological diagnosis.

### Integrated imaging evaluation

LDCT remains the reference imaging modality for lung cancer screening because randomized trials have demonstrated reductions in lung cancer mortality in appropriately selected high-risk populations, and population-level analyses have associated LDCT implementation with a change in stage and improved survival.^1,13,67^ For pulmonary nodule evaluation, acquisition and reconstruction parameters are particularly important, especially for nonsolid nodules and PSNs, for which the nodule size and the solid component may be underestimated on thicker sections. The NCCN Lung Cancer Screening Guidelines recommend a slice thickness of ≤1.5 mm; I-ELCAP recommends a reconstructed slice thickness of ≤1.25 mm; and the 2024 Chinese recommendations suggest 1.00–1.25 mm reconstruction, no interval when the reconstruction thickness is ≤0.625 mm, and a reconstruction interval not exceeding 80% of the slice thickness when 1.00–1.25 mm reconstruction is used.^12,50,68^ These technical parameters are important for improving the detection and follow-up assessment of small and subsolid nodules (SSNs). Health economic analyses across diverse health care systems also support the cost-effectiveness of LDCT screening, but the economic value depends on local epidemiology, eligibility criteria, screening costs, downstream work-up, and willingness-to-pay thresholds. Reported incremental cost-effectiveness ratios (ICERs) for LDCT lung cancer screening include US $49,200/quality-adjusted life-years (QALY) for the NLST criteria, US $68,600/QALY for the CMS criteria, US $96,700/QALY for the USPSTF criteria in the United States,^69^ NZ $34,400/health-adjusted life-years (HALY) in New Zealand,^70^ €3,297/QALY in Italy,^71^ and CA $20,724/QALY in Canada.^72^ These variations highlight the importance of adapting screening strategies and economic evaluations to local health care contexts. These findings support using LDCT as the backbone of lung cancer screening in eligible populations and indicate that screening intervals and eligibility criteria should be tailored to local risk distributions, resource capacity, and implementation approaches. Within this CT-based framework, CE-CT, particularly DCE-CT, may provide complementary information after nodule detection by characterizing enhancement behavior and vasculature-related features. Previous multicenter evidence has shown that the absence of significant enhancement is strongly associated with benignity, supporting the diagnostic relevance of enhancement-based assessment in selected solid indeterminate nodules.^73^ More recent comparative evidence has evaluated DCE-CT alongside PET/CT for the characterization of solitary pulmonary nodules, suggesting that DCE-CT may have a role as a complementary problem-solving tool, particularly when PET/CT is unavailable or when enhancement characteristics are clinically informative.^74^ However, CE-CT should not be considered a replacement for LDCT screening or PET/CT-based metabolic assessment because it addresses a different biological dimension of nodule characterization. Accordingly, CE-CT is best positioned as a selective adjunct within the broader CT-centered diagnostic approach rather than as a standalone screening modality.

MRI is not a first-line modality for lung cancer screening but has been investigated as a radiation-free adjunct in selected follow-up or surveillance contexts.^75^ Meta-analytic and early clinical data indicate that MRI performance is strongly size dependent, with sensitivity approaching that of CT mainly for nodules ≥8–10 mm and remaining lower for smaller lesions.^75,76^ Diffusion-weighted MRI may provide additional value for benign–malignant discrimination, although the current evidence is heterogeneous and largely diagnostic rather than screening-based.^77,78^ MRI is more defensible in longitudinal management than in initial population-based detection.^79–81^ In a multicenter follow-up study of incidental pulmonary nodules, particularly in patients undergoing longitudinal CT-based follow-up, MRI detected the most persistent nodules identified on follow-up LDCT and showed high agreement with LDCT for interval growth assessment and patient-level Lung-RADS categorization.^81^ Clinical adoption remains limited by lower sensitivity for small nodules, ground-glass lesions, calcified nodules, and lesions near the pleura or hilum.^80,82^ Motion artifacts, protocol heterogeneity, scanner accessibility, and lack of standardization further limit its generalizability.^76^ Because MRI is also time- and resource-intensive, prospective cost-effectiveness evidence supporting its use in population-based lung cancer screening remains limited.^76^ Although modeling analyses suggest that MRI-based screening may reduce false-positive results while providing life expectancy gains comparable to those of LDCT, these findings require prospective validation.^83^ Accordingly, MRI should be regarded as a selective radiation-free adjunct for follow-up or surveillance in carefully selected contexts rather than as a replacement for LDCT in population-based lung cancer screening.

FDG PET/CT is primarily a postdetection tool for malignancy risk stratification, biopsy planning, and staging rather than a screening modality, with its greatest utility demonstrated for indeterminate nodules larger than 8 mm or those with a sufficiently large solid component.^84–86^ PET-derived parameters, including standardized uptake value metrics, metabolic tumor volume, and total lesion glycolysis, may further refine risk assessment in certain contexts.^84^ However, FDG PET/CT has several well-recognized limitations. Benign inflammatory or infectious lesions may show increased FDG uptake, reducing specificity, whereas indolent tumors or those with low metabolic activity may produce false-negative results.^84,85^ PET/CT is less reliable for detecting pGGNs, nodules with a small solid component, and lesions in regions where infectious or inflammatory lung disease is prevalent.^12,87,88^ Emerging tracers may improve their postdetection applications in certain contexts.^89,90^ At present, however, these approaches remain more relevant to risk stratification and preoperative evaluation than to screening, and prospective validation is still needed before routine clinical adoption.

Overall, LDCT remains the backbone of lung cancer screening and pulmonary nodule surveillance, whereas CE-CT serves as a selective CT-based adjunct for the enhancement-related characterization of indeterminate nodules. MRI and PET/CT play additional complementary roles in radiation-free follow-up, malignancy risk assessment, biopsy planning, and staging.

### Nonsurgical tissue diagnosis

CT-guided transthoracic needle biopsy (TTNB) remains a key nonsurgical diagnostic approach for peripheral pulmonary lesions, although its diagnostic accuracy significantly decreases with decreasing nodule size.^91^ Bronchoscopic approaches provide an alternative route for cytologic or histologic diagnosis, particularly when percutaneous biopsy is undesirable or when airway access is favorable.^92–95^ Radial endobronchial ultrasound (EBUS)-guided transbronchial biopsy improves lesion localization and is more sensitive and accurate than conventional transbronchial biopsy for malignant peripheral lesions.^92^ Electromagnetic navigation bronchoscopy (ENB) and virtual bronchoscopic navigation (VBN) extend access to peripheral lesions beyond the reach of conventional bronchoscopy, although the yield remains influenced by airway anatomy, lesion size, bronchus sign, cost, and operator experience.^92–94^ Meta-analytic evidence supports the role of guided bronchoscopy, with higher diagnostic yield in nodules >20 mm and in lesions with a bronchus sign.^93^ Robot-assisted bronchoscopy may improve distal airway access, catheter stability, and lesion targeting, but much of the comparative evidence is early, platform-based, or preclinical, and larger real-world studies are still needed.^95–97^

### Preoperative localization

Preoperative localization techniques should be considered separately from biopsy, as they do not provide a tissue diagnosis. Their purpose is to facilitate the resection of small, deep, or impalpable nodules during minimally invasive surgery. Hook wire localization is technically straightforward and characterized by high success rates, but the risks of pneumothorax, hemorrhage, and wire dislodgement, together with the need for prompt surgery after placement, limit its use.^98–100^ Microcoil localization offers greater scheduling flexibility and may reduce dislodgement-related problems, although it is more technically demanding.^100–102^ Liquid marker localization, including indocyanine green-based approaches, is relatively simple and useful for selected superficial nodules, but diffusion, limited visibility, local irritation, and material-related costs may restrict its broader application.^103^ CT-based three-dimensional reconstruction and patient-specific guide-assisted approaches may improve localization accuracy and procedural planning, but cost and positioning requirements remain practical barriers.^104^ Augmented reality-guided navigation remains investigational, with early evidence from preclinical studies but limited routine clinical adoption.^105,106^

## Noninvasive biomarker-assisted risk stratification: liquid biopsy

**Fig. 2:**
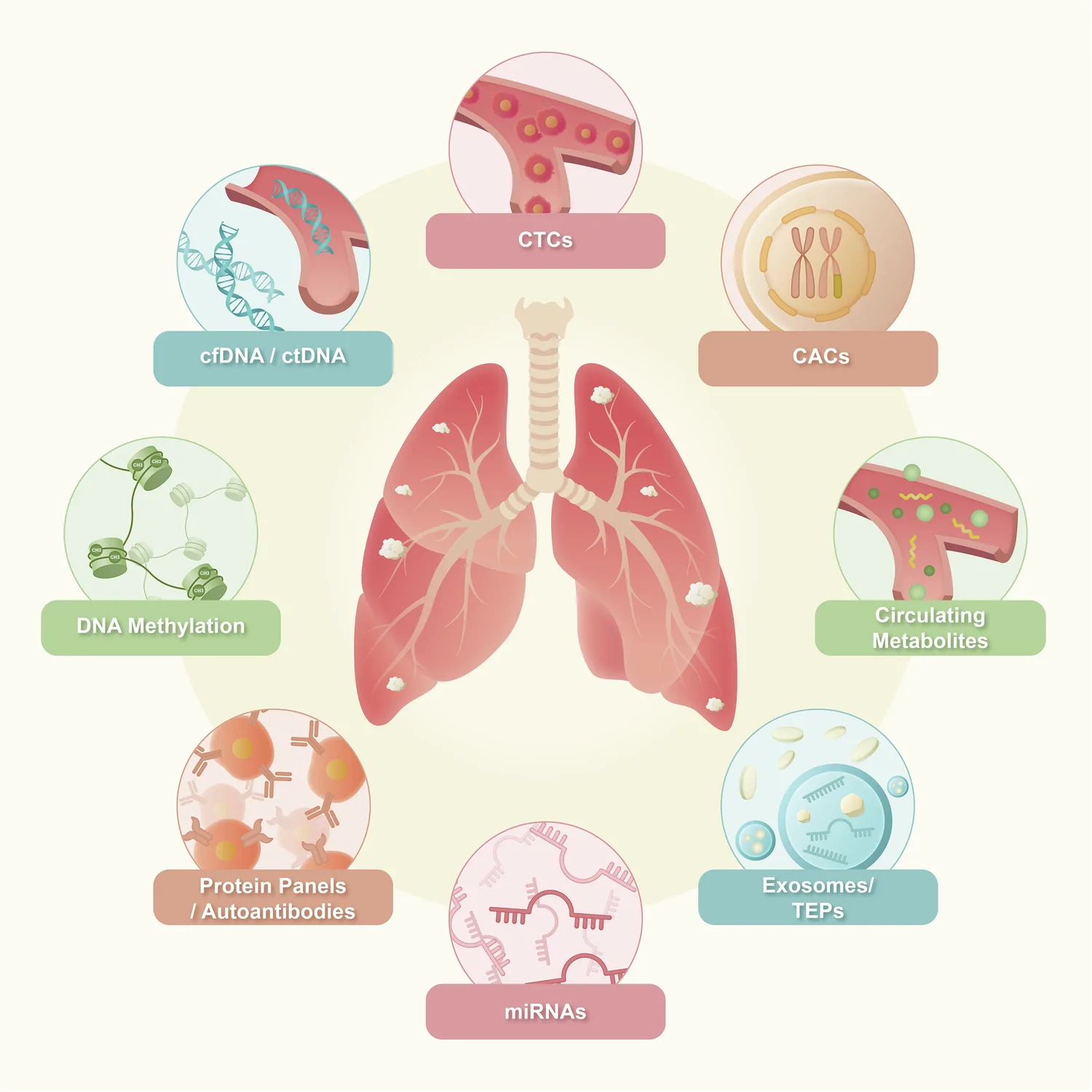
Major liquid biopsy analytes evaluated for pulmonary nodule risk assessment. Representative liquid biopsy analytes include cell-free DNA and circulating tumor DNA, circulating tumor cells, circulating genetically abnormal cells, microRNAs, DNA methylation signatures, circulating metabolites, protein panels and autoantibodies, extracellular vesicles such as exosomes, and tumor-educated platelets. These analytes differ in their biological origin, abundance, analytical requirements, and intended clinical use. In patients with imaging-detected pulmonary nodules, liquid biopsy is most appropriately investigated as an adjunct for risk refinement in defined populations, rather than as a stand-alone diagnostic test or as a replacement for computed tomography, longitudinal imaging, or tissue diagnosis when indicated. cfDNA cell-free DNA, CACs circulating genetically abnormal cells, CTCs circulating tumor cells, ctDNA circulating tumor DNA, miRNAs microRNAs, TEPs tumor-educated platelets. Created using Adobe Illustrator

Liquid biopsy has emerged as a broad noninvasive oncological approach.^107,108^ It allows the assessment of circulating biological signals, enabling the potential detection of tumor-associated biomarkers in plasma and other body fluids such as saliva, urine, cerebrospinal fluid, and sputum. Many circulating analytes are thought to be released into body fluids through apoptosis, necrosis, and active secretion.^107,108^ On the basis of biological origin, liquid biopsy analytes can be broadly classified into four categories: (1) cell-free nucleic acid alterations, including alterations in cell-free DNA (cfDNA), circulating tumor DNA (ctDNA), microRNAs (miRNAs), and DNA methylation signatures; (2) intact cellular components, mainly circulating tumor cells (CTCs) and circulating genetically abnormal cells (CACs); (3) soluble proteins, metabolites, and related molecules; and (4) extracellular vesicles (EVs), particularly exosomes. Compared with tissue biopsy, liquid biopsy is minimally invasive, repeatable, and suitable for serial assessment. However, the low tumor burden of many early lung cancers and indeterminate pulmonary nodules limits assay sensitivity and clinical interpretability. In pulmonary nodule care, risk enrichment before or alongside lung cancer screening, postimaging risk refinement for indeterminate nodules, and longitudinal biological assessment in selected contexts are most plausible. Liquid biopsy should be viewed as a complementary approach to CT-based evaluation and tissue diagnosis when indicated, rather than as an independent diagnostic approach or replacement for established management protocols. The principal classes of liquid biopsy analytes evaluated in pulmonary nodule assessment are summarized in Fig. 2.

The clinical value of liquid biopsy depends on both the analyte and the intended use setting. Some assays may be evaluated as blood-based adjuncts to lung cancer screening, whereas others are better positioned for post-imaging assessment of CT-detected pulmonary nodules, particularly when integrated with radiological features and clinical risk factors.

From a translational perspective, liquid biopsy should not be judged solely by AUC, sensitivity, or specificity, but by whether it improves decision-making at defined clinical points, such as risk enrichment before LDCT, restratification of indeterminate nodules, avoidance of unnecessary invasive procedures, or selection of patients requiring closer follow-up.^109^ Because liquid biopsy biomarkers differ in terms of analyte type, assay platform, and validation population, their clinical contribution is necessarily context dependent. In guideline-based management, solid indeterminate pulmonary nodules measuring >8 mm and ≤30 mm with an estimated pretest malignancy probability of 5%–65% represent a clinically uncertain decision range in which CT surveillance, PET/CT, nonsurgical biopsy, or surgical evaluation may be considered according to imaging findings, estimated risk, patient fitness, and preferences.^9^ Intended use populations remain assay-specific because existing studies differ substantially in patient nodule size, baseline malignancy probability, detection setting, analyte, and assay platform. At present, liquid biopsy is best viewed as a complementary biological layer within CT- and risk model-based approaches rather than as an independent route to rule out malignancy, initiate surgery, or discontinue guideline-directed surveillance.^109^

### Circulating tumor cells

CTCs are important components of liquid biopsy that are linked to invasive and metastatic processes; epithelial‒mesenchymal transition (EMT) may facilitate their dissemination and phenotypic plasticity.^110^ CTCs have been reported in some early-stage lung cancer cohorts, but their detection in screening populations remains inconsistent. The epithelial cell adhesion molecule (EpCAM)-based CellSearch® system has been approved by the Food and Drug Administration (FDA) for CTC enumeration to monitor patients with certain metastatic cancers, including breast, colorectal, and prostate cancer, but not for pulmonary nodule triage or lung cancer screening; EpCAM downregulation during EMT may limit the sensitivity of this system.^111^

Several small- to moderate-sized studies have evaluated CTC-based assays for early lung cancer detection and pulmonary nodule assessment, mostly in selected or enriched cohorts rather than in unselected screening populations. A multicenter study suggested that folate receptor-positive CTCs (FR+CTCs) were associated with malignancy and invasion-related assessment in indeterminate lung nodules, particularly when their detection was combined with tumor diameter.^112^ For nodules <2 cm, the combination of CT and CTC detection increased the sensitivity and specificity to 89.9% and 83.9%, respectively, in the reported cohort.^113^

Several limitations have limited enthusiasm for routine CTC-based testing, including small sample sizes in many studies, interplatform heterogeneity, variable sensitivity in early-stage disease, and limited integration with standardized imaging-based risk models. More recently, CTC-derived RNA testing has broadened this field. Wang et al. developed a multiantibody CTC capture and reverse-transcription droplet digital PCR model, termed the non-small cell lung cancer (NSCLC) CTC ScoreD, which achieved an AUC of 0.93 for distinguishing early NSCLC from benign pulmonary nodules in the reported early-stage cohorts.^114^ In contrast, in the AIR prospective cohort study designed for screening, the use of ISET-CTC in a high-risk chronic obstructive pulmonary disease (COPD) population achieved a sensitivity of only 26.3% despite a specificity of 96.2%, suggesting that CTCs remain of limited value as a stand-alone screening tool.^115^ Conversely, certain cytogenetic cell-based platforms have been reported to have better post-CT triage performance. A 4-color fluorescence in situ hybridization (FISH) study in patients with LDCT-detected nodules reported a sensitivity of 89% and a specificity of 100% for differentiating benign from malignant nodules, suggesting the potential value of certain platforms for post-CT nodule triage.^116^

Taken together, the results of CTC studies support biological plausibility and potential adjunctive value after CT, particularly when CTC readouts are integrated with tumor diameter or imaging findings.^112,113^ Nevertheless, current evidence remains heterogeneous across assay platforms and certain CT-suspicious, LDCT-detected, preoperative, or pathology-enriched cohorts, with no reproducible nodule size or malignancy probability window established across CTC methods.^115,116^ Thus, CTC assays are currently best positioned as investigational or adjunctive tools for indeterminate nodules to be interpreted alongside clinical variables or imaging findings rather than as standalone diagnostic solutions for lung cancer screening or routine pulmonary nodule triage. Trial registrations indicate continued evaluation of CTC-based and combined liquid biopsy models for pulmonary nodule management, underscoring translational interest in this field (NCT06187935, NCT02653859, and NCT04156360).

### Cell-free DNA and circulating tumor DNA

cfDNA is released into body fluids primarily via apoptosis and necrosis, whereas the tumor-derived fraction, ctDNA, is associated with genomic alterations, including mutations and insertions/deletions.^117,118^ As a circulating fragment of the tumor genome, ctDNA has attracted interest for early lung cancer detection and malignancy assessment of certain resectable or CT-detected lung lesions.^119^

Approaches for cfDNA/ctDNA analysis that have been explored in early cancer detection research include digital PCR-based assays for validation; targeted error-corrected sequencing, such as TEC-Seq; CAPP-Seq with integrated genomic features; and genome-wide fragmentomic profiling, such as DELFI. Methylation-based methods have also been investigated for determining the cfDNA tissue of origin.^118,120–122^ In a surgical cohort of patients with operable lung lesions, ultradeep targeted sequencing of plasma ctDNA mutations achieved ~69% sensitivity and 96% specificity for distinguishing malignant from benign lesions.^119^ In studies using quantitative polymerase chain reaction to assess total plasma cfDNA, Szpechcinski et al. reported 90.0% sensitivity and 80.5% specificity for discriminating patients with resectable NSCLC from healthy controls at a reported cutoff of 2.8 ng/mL,^123^ whereas the cfDNA concentration distinguished patients with NSCLC from those with benign pulmonary nodules and healthy controls with 86.4% sensitivity and 61.4% specificity.^124^ In an early lung cancer detection study, cfDNA fragmentation features combined with clinical risk factors and CEA, followed by CT imaging, detected 94% of cancers with 80% specificity.^125^ These studies support biological plausibility and technical feasibility, but their populations and endpoints differ from those of routine post-CT management of indeterminate pulmonary nodules. cfDNA/ctDNA testing remains constrained by a low tumor fraction, short half-life, and reduced sensitivity in individuals with early-stage or low-volume disease.^118,126^ Consistent with this observation, even advanced error-suppressed sequencing strategies such as TEC-Seq and CAPP-Seq/Lung-CLiP have reported variable (and often modest) ctDNA sensitivity in early-stage NSCLC cohorts. These technically refined early detection approaches were evaluated in selected populations and should not be interpreted as validated pulmonary nodule triage tools.^120,121^ Genome-wide cfDNA fragmentation analysis, exemplified by the DELFI approach, and multianalyte blood tests, such as CancerSEEK and DETECT-A, demonstrate the broader feasibility of blood-based early cancer detection, but these studies provide screening or multicancer early detection evidence rather than direct pulmonary nodule triage data.^122,127,128^

Beyond mutation profiling, cfDNA-based research has expanded into fragmentomics and related analytic dimensions. Compared with conventional mutation-based ctDNA approaches, these methods may capture complementary biological signals in patients with early-stage disease and low-tumor-burden lesions and are increasingly being evaluated both as adjuncts to LDCT and as postimaging risk-stratification tools. In a clinical validation study aimed at increasing lung cancer screening in an LDCT-eligible population, a low-coverage whole-genome cfDNA fragmentome assay achieved a stage-weighted sensitivity of 80% and a specificity of 58%, supporting its potential use as an adjunct rather than a replacement for LDCT.^129^ After pulmonary lesions have been identified by imaging, cfDNA-based models have been used more often for malignancy risk stratification. Peng et al. developed a plasma-circulating cell-free mitochondrial DNA fragmentomics model that distinguished individuals with NSCLC from those with benign pulmonary disease, with AUCs above 0.95 across validation datasets and an AUC of 0.9151 for lesions ≤1 cm; the reported decision analyses suggested, but did not prove, potential reductions in unnecessary invasive procedures.^130^ Similarly, Zhao et al. integrated clinical variables, deep learning-based CT radiomics, and cfDNA fragmentomic features derived from 5-methylcytosine-enriched regions for indeterminate pulmonary nodules. Compared with unimodal models, the clinic-RadmC model achieved an AUC of 0.923 in the external test cohort and showed more favorable decision curves and model-based clinical utility.^131^ Another epigenome-guided fragmentomics model, developed mainly in an early lung cancer detection context, achieved an AUC of 0.94, a sensitivity of 90.4%, and a specificity of 83.1% in an independent validation cohort, with encouraging performance in stage I disease and microinvasive adenocarcinoma; however, enrichment for suspicious malignant lesions and limited benign nodule representation may restrict generalizability to real-world indeterminate nodule management.^132^ The ASCEND-LUNG proof-of-concept study evaluated a comprehensive lung cancer screening and postscreening pulmonary nodule management system; its AI-aided pulmonary nodule diagnostic model incorporated cfDNA methylation and CT image features and achieved a sensitivity of 81.1% and a specificity of 76.0% in the reported independent validation cohort.^133^ Collectively, these studies support integrated imaging–molecular risk stratification as a promising research direction, but prospective evidence showing improved patient outcomes or safer de-escalation remains limited.

Several clinical studies (NCT04182893, NCT03320044, NCT05171491, and NCT05495672) are evaluating cfDNA-, ctDNA-, exosome-, and multiomics-based approaches to evaluate pulmonary nodules, but trial registry information should be used to indicate ongoing clinical translation rather than to support diagnostic performance claims without peer-reviewed results. Overall, cfDNA-based approaches may be most appropriately framed as adjunctive tools in two related contexts. First, fragmentome-based blood tests have been evaluated for risk enrichment before or alongside LDCT, including the DELFI approach reported by Mathios et al. and the clinically validated cfDNA fragmentome assay reported by Mazzone et al.; the intended complementary role of such testing relative to LDCT and clinical risk models was clarified in a subsequent reply.^125,129,134^ Second, for indeterminate pulmonary nodules detected on CT, integrated models combining cfDNA methylation or 5mC-enriched fragmentomic features with clinical and radiological/radiomic variables have shown improved benign–malignant discrimination in multi-institutional studies, including those by Zhao et al.^131,133^ and ASCEND-LUNG. Given the heterogeneity of diagnostic cohorts, including certain operable lung lesions to NSCLC, benign lung disease, and healthy control cohorts with variable lesion sizes, the current evidence does not yet define a universal nodule size threshold or support the replacement of CT-based approaches with blood-only algorithms for biopsy, surgery, or the termination of surveillance.^119,130^

### DNA methylation

DNA methylation has been extensively investigated as an epigenetic liquid biopsy biomarker. Because some methylation alterations may occur early in tumorigenesis and are relatively stable, methylation-based assays have been explored for early lung cancer detection and adjunctive post-CT pulmonary nodule or lung disease risk assessment. Early studies of SHOX2, RASSF1A, PTGER4, and multigene methylation panels indicated their diagnostic value, although not all evidence was generated in plasma or pulmonary nodule triage contexts. For example, combined SHOX2 and RASSF1A promoter methylation analysis achieved a sensitivity of 74.07% and a specificity of 100% for detecting early lung adenocarcinoma in FFPE tumor samples.^135^ Although these tissue-based results do not directly establish plasma performance, they support the biological relevance of SHOX2/RASSF1A methylation markers. In a hypothesis-generating retrospective dataset cited in the protocol for a prospective 10,560-patient multicenter trial, a ctDNA methylation classifier achieved a sensitivity of 82.5% and a specificity of 83.3% for distinguishing malignant from benign pulmonary nodules.^136^ In addition, a combined SHOX2/PTGER4 model yielded an AUC of 0.88 for distinguishing lung cancer patients from those with nonmalignant lung disease or healthy controls and reached a sensitivity of 67% with 90% specificity.^137^

More directly relevant evidence comes from integrated methylation-based models developed for pulmonary nodule risk stratification. He et al. developed and externally validated PulmoSeek Plus, which integrates clinical characteristics, imaging markers, and plasma cfDNA methylation biomarkers to assess the risk of 5–30 mm pulmonary nodules. In the independent validation cohort, the model achieved an AUC of 0.91 and maintained favorable performance for early-stage nodules, small nodules, and SSNs. Its dual-threshold strategy classified nodules into low-, intermediate-, and high-risk groups, and model-based estimates suggested potential reductions in unnecessary surgeries and delays in treatment, rather than proven improvements in clinical outcomes.^138^ In the prospective multicenter LUNG-TRAC study, a ctDNA methylation model outperformed traditional risk stratification models in the validation cohort, with an AUC of 0.810, sensitivity of 74.4%, and specificity of 73.7%; however, the performance was lower in the multicenter test set, indicating the need for further external validation.^139^

Liang et al. developed a simplified model that combines plasma cfDNA methylation, age, and five CT features to stratify pulmonary nodule risk. In the external validation cohort, the integrated model achieved an AUC of 0.89, outperforming both the methylation-only model and conventional clinical tools, including the Mayo Clinic and Brock models. Its dual-threshold design provided a practical framework for surveillance, intensified evaluation, or invasive intervention according to estimated risk.^140^

Overall, cfDNA/ctDNA methylation is emerging as a nodal-oriented liquid biopsy strategy for post-CT risk stratification, with integrated clinical CT methylation models showing stronger risk classification than methylation-only approaches do.^138–141^ However, current evidence is largely derived from selected CT-detected, surgically evaluated, or solitary noncalcified 5–30 mm nodules rather than unselected all-nodule populations. These models therefore support molecular imaging risk refinement and potential decision support, but prospective management impact studies are still needed to determine whether they improve outcomes or reduce unnecessary invasive procedures.^138–141^

### Protein panels and autoantibodies

Protein- and autoantibody-based assays remain attractive for translational evaluation because many are compatible with established immunoassay workflows and routine blood sampling. Conventional marker panels incorporating proteins such as CEA, CYFRA21-1, CA125, SCC, ProGRP, or related analytes have shown only modest standalone performance, particularly when applied to early detection or externally validated nodule risk models. For example, Mazzone et al. reported 49% sensitivity at 96% specificity for serum protein/autoantibody panels in an independent test set, whereas the LCBP nodule model showed substantially weaker discrimination in the validation dataset than in the training dataset.^142,143^ More recent plasma protein or proteomic classifiers have been evaluated as adjuncts to clinical and radiologic risk assessment rather than replacements for CT-based approaches. The C4c/CYFRA21-1/CRP and pro-SFTPB/CA125/CYFRA21-1/CEA panels improved risk discrimination in selected pulmonary nodule cohorts, whereas the PANOPTIC classifier was designed mainly as a rule-out adjunct for low-to-intermediate risk nodules.^144–146^

Multimarker protein panels may improve discrimination when they are integrated with clinical or imaging-based risk models. The INTEGRAL program was designed to systematically evaluate circulating protein biomarkers for pre-LDCT risk prediction and post-LDCT benign versus malignant nodule classification.^147^ Blood-based protein and protein–autoantibody panels, including combinations of CEA, CYFRA21-1, CA125, HGF, NY-ESO-1 autoantibodies, C4c, CRP, pro-SFTPB, ProGRP, and SCC, have shown variable but potentially additive performance for early lung cancer detection or pulmonary nodule risk stratification.^142–145^ Recent proteomic and circulating protein biomarker studies have identified blood-based protein signatures associated with pulmonary nodule malignancy, imminent lung cancer diagnosis, and lung cancer risk, but their role in routine decision-making still requires independent clinical validation, workflow assessment, and health economic evaluations.^148–150^

PANOPTIC evaluated a clinically defined nodule population, focusing on patients aged ≥40 years with 8–30 mm nodules and a clinician-assessed probability of cancer (pCA) ≤ 50%. An integrated classifier combining LG3BP, C163A, and five clinical/imaging factors achieved high sensitivity and negative predictive value (NPV), supporting its potential role as a rule-out adjunct for certain low-to-moderate risk nodules.^146^ Two-year PANOPTIC follow-up data confirmed the stability of likely benign classifications and real-world decision impact data associated with classifier use with fewer invasive procedures and clinical visits in patients with a pCA ≤50%.^151,152^ Together, these studies define a relatively concrete evidence window for plasma protein classifiers as adjunctive rule-out tools in selected patients with low- to moderate risk pulmonary nodules, although clinical decisions should still be integrated with CT images, guideline approaches, patient fitness, and longitudinal follow-up.

Tumor-associated autoantibodies are circulating antibodies directed against tumor-derived or aberrantly expressed proteins, and their relative stability and early emergence before clinical diagnosis have supported their evaluation as adjunctive biomarkers for lung cancer detection.^153,154^ In the ECLS randomized trial, the seven autoantibody EarlyCDT-Lung assay, followed by imaging in participants with positive test results, showed high specificity but limited sensitivity and was associated with a lower rate of late-stage lung cancer diagnosis.^155^ In pulmonary nodule cohorts, EarlyCDT-Lung positivity increased the positive predictive value of CT-based assessment and nodule-based risk models, particularly in threshold analyses with high specificity.^156^ In a Chinese multicenter validation study, a seven-autoantibody panel showed diagnostic performance for early-stage NSCLC and increased the positive predictive value of CT-based evaluation, including among patients with CT-detected ground-glass nodules and/or solid nodules.^157^ Overall, autoantibody assays appear better suited for improving specificity and supporting postimaging risk assessment than for replacing imaging-based approaches.

### MicroRNAs

Circulating miRNAs in serum, plasma, or peripheral blood cells have been evaluated as minimally invasive biomarkers across several lung cancer-related contexts. Early-stage NSCLC detection studies have reported serum levels of miR-1254 and miR-574-5p and plasma panels that include miR-20a, miR-145, miR-21, miR-223, and miR-221.^158,159^ In LDCT screening cohorts, plasma or blood miRNA classifiers have been evaluated for lung cancer risk prediction and screening interval personalization.^160,161^ With respect to CT-detected pulmonary nodules, some studies have investigated miRNA-based models as adjuncts to radiological and clinical assessments.^162,163^ In one study of individuals with pulmonary nodules, a peripheral blood mononuclear cell (PBMC)-derived model combining miR-19b-3p, miR-29b-3p, nodule spiculation, and smoking pack-years yielded an AUC of 0.91 for malignant versus benign nodule discrimination in the training and testing datasets.^163^ Overall, these data remain insufficient for direct clinical generalization because many studies were small, case‒control, or platform-specific, and early detection findings should not be assumed to establish utility for CT-detected nodule triage. Related non-miRNA RNA biomarkers, such as circulating circRNAs and tsRNAs, should be discussed separately as complementary RNA-based approaches.^164,165^

From a clinical perspective, miRNA-based assays may serve either as blood-based adjuncts within lung cancer screening workflows or as tools for further risk assessment after imaging positivity, and these intended use contexts should be distinguished. Bianchi et al. initially described a serum-circulating miRNA diagnostic test to identify asymptomatic high-risk individuals with early-stage lung cancer, and Montani et al. subsequently refined this approach to develop the 13-miRNA miR-Test, which was validated in the COSMOS high-risk screening cohort.^166,167^ In the independent validation set of 1008 COSMOS participants, the miR-Test achieved an AUC of 0.85, an accuracy of 74.9%, a sensitivity of 77.8%, and a specificity of 74.8%.^167^ Across all CT screening cohorts comprising 1,115 individuals, the reported sensitivity and specificity were 79.2% and 75.9%, respectively, with a negative predictive value >99%.^167^ In high-risk COPD populations, circulating miR-1246 and miR-206 levels have been evaluated as candidate biomarkers for early lung cancer detection or risk enrichment, although their use in prospective screening workflows requires further validation.^168^

Several studies have evaluated miRNA assays in conjunction with LDCT, which is more directly relevant to screening implementation. In MILD-related studies, a plasma miRNA signature classifier combined with LDCT reduced the false-positive rate of LDCT from 19.4% to 3.7%, suggesting its potential value in limiting unnecessary downstream examinations.^160^ In the BioMILD trial, combined CT/microRNA signature classifier (MSC) stratification revealed markedly different 4-year lung cancer incidences across risk groups: 0.8% in CT − /MSC − , 1.1% in CT − /MSC + , 10.8% in CT + /MSC − , and 20.1% in CT + /MSC+ participants.^161^ More recently, Sozzi et al. evaluated an upfront blood miRNA test in individuals reluctant to undergo LDCT; the assay identified 58.7% of cancers and 56.5% of early-stage cases that would otherwise have been detected by standard LDCT screening, suggesting a potential role in improving screening participation or triaging LDCT-reluctant individuals rather than replacing standard LDCT screening.^169^

After LDCT detection of pulmonary nodules, blood-based molecular or immune assays may provide adjunctive risk stratification rather than serving as stand-alone diagnostic replacements. A whole-blood 41-gene expression classifier achieved an independent validation AUC of 0.825 for benign versus malignant nodules detected by LDCT and showed 64% specificity with 90% sensitivity in 8–14 mm nodules.^170^ A multicenter retrospective tsRNA study developed a five-tsRNA/CT-feature nomogram for single nodules ≤2 cm, with AUCs of 0.930 and 0.943 in the internal and external validation cohorts, respectively.^165^ In BioMILD participants with suspicious LDCT findings, a combined plasma MSC and PBMC-based immune classifier achieved a sensitivity of 0.96 and NPV of 0.98 for cancer patients diagnosed within 2 years, supporting further evaluation of blood-based rule-out strategies after positive LDCT findings.^171^

Overall, circulating miRNAs have been investigated in both lung cancer screening and post-CT pulmonary nodule evaluation, but these applications represent distinct intended use contexts. In CT-detected nodule cohorts, serum-, plasma-, or PBMC-based miRNA models have been evaluated for malignant versus benign discrimination.^162,163,172^ In contrast, the serum miR-Test was developed for risk enrichment among high-risk screening populations, whereas the MILD/MSC and BioMILD data support LDCT adjunct-based risk stratification and screening interval personalization.^160,161,167^ Consistent with American Thoracic Society guidance that clinical utility requires evidence of improved management or outcomes, current data support the use of miRNA assays primarily as complementary risk refinement tools rather than as stand-alone algorithms for biopsy, surgery, or surveillance termination decision-making.^109^

### Other noninvasive molecular and body-fluid biomarkers

In addition to the major analytes discussed above, other noninvasive molecular biomarkers detectable in blood or other body fluids may provide complementary information for lung cancer detection and evaluation of pulmonary nodules. As liquid biopsy biomarkers, exosomes and EVs are attractive for their relative biological stability and accessibility in body fluids, although preanalytical variation, isolation methods, and analytical standardization remain important barriers to clinical translation. Zhang et al. reported that serum exosomal miR-5684 and miR-125b-5p levels distinguished NSCLC patients from healthy donors with an AUC of 0.793, a sensitivity of 82.7%, and a specificity of 62.1%; for early-stage NSCLC patients versus healthy donors, the corresponding AUC, sensitivity, and specificity were 0.744, 80.6%, and 60.9%, respectively.^173^ In addition to being directly relevant to small nodule-associated early lung cancer detection, EV-based protein and RNA panels have shown encouraging diagnostic performance. Han et al. developed an EV membrane protein panel, EVELC-M5, which achieved an AUC of 0.926 in the validation cohort and, in a subgroup analysis rather than a management impact study, distinguished benign nodules ≤8 mm from lung cancer nodules of the same size with an AUC of 0.931.^174^ Similarly, Meng et al. established a five-RNA signature derived from plasma small EVs in patients with indeterminate pulmonary nodules detected by LDCT with subsequent definitive classification; this signature distinguished T1 lung adenocarcinoma lesions from benign pulmonary nodules with AUCs of 0.971 and 0.950 in the validation and prospective test cohorts, respectively, and showed stronger discrimination than the CT-based comparator used in that study.^175^ Although these results are promising, EV-based assays remain limited by platform standardization, cohort selection, and uncertain incremental value beyond CT-based assessment.

Tumor-educated platelets (TEPs), which are platelets altered by tumor-related systemic signals, have also been explored as blood-based biomarkers. In a pancancer study that included NSCLC samples, rather than a lung nodule-specific triage cohort, Best et al. reported that TEP mRNA profiles distinguished cancer patients from healthy donors with a sensitivity of 97.0%, a specificity of 94.0%, and an accuracy of 96.0%.^176^ Platelet ITGA2B expression showed promising diagnostic performance for early NSCLC; for stage I NSCLC lesions versus benign pulmonary nodules and healthy controls, ITGA2B achieved an AUC of 0.940, a sensitivity of 96.4%, and a specificity of 78.6% in the test cohort but lower performance in the validation cohort, with an AUC of 0.842, a sensitivity of 87.8%, and a specificity of 56.5%.^177^ These data support further evaluation of platelet-derived biomarkers but do not yet establish their role in routine pulmonary nodule triage.

Host-response biomarkers measured in blood also appear relevant to the evaluation of pulmonary nodules. Søgaard et al. reported that blood T-cell receptor (TCR) repertoire profiling provided independent diagnostic information beyond age, smoking history, and nodule size, and improved risk prediction in individuals with indeterminate lung cancer risk, derived from a lung cancer screening cohort and from individuals evaluated for abnormal chest CT findings.^178^ However, because TCR repertoire features may be shaped by the human leukocyte antigen (HLA) background, immune status, infection history, and nonneoplastic inflammatory conditions, these assays are currently better regarded as adjunctive host response biomarkers than stand-alone pulmonary nodule classifiers.

Metabolomic alterations can also be detected in urine, blood, saliva, and other body fluids under pathological conditions such as cancer, although current metabolomic evidence in lung cancer is derived from risk association studies rather than validated pulmonary nodule triage assays.^179^ Patients with lung cancer exhibit alterations in amino acid and energy metabolism, with branched-chain amino acids such as valine, leucine, and isoleucine repeatedly being implicated in lung cancer-associated metabolic profiles.^180^ Technologies such as gas chromatography‒mass spectrometry, liquid chromatography‒mass spectrometry, and nuclear magnetic resonance spectroscopy, together with analysis platforms such as MetaboAnalyst, are typically used for metabolic profiling and pathway interpretation.^180^ In a preliminary salivary metabolomics study designed to differentiate lung cancer lesions from benign lung lesions, a combination of four metabolites, namely, diethanolamine, cytosine, lysine, and tyrosine, yielded an AUC of 0.729 (*P* = 0.003), whereas the tryptophan level was significantly reduced in lung cancer samples (AUC = 0.663, *P* = 0.036).^181^ These noninvasive biomarkers may provide supplementary information upon integration into existing pulmonary nodule diagnostic workflows, but important limitations, including small sample sizes, lack of external validation, incomplete metabolite identification, and insufficient standardization, remain.^180,181^ At present, metabolomic assays should be viewed as exploratory rather than clinically actionable tools for the management of pulmonary nodules.

CACs represent another emerging liquid-biopsy biomarker with potential relevance to the diagnosis of pulmonary nodules. FISH-detectable abnormalities on chromosomes 3 and 10, including probes targeting 3p22.1/3q29 and 10q22.3/CEP10, have been used to detect CAC and evaluate early lung cancer and pulmonary nodules.^182–184^ In a prospective cohort of patients with ≤30 mm pulmonary nodules, a 4-color FISH CAC assay showed diagnostic discrimination in both the training and validation cohorts, with reported AUCs of 0.887 and 0.823, respectively.^182^ In the more challenging subgroup of ≤10 mm nodules, CACs achieved a sensitivity of approximately 70.5% and a specificity of 86.4%, and integrated models incorporating CACs and clinical factors improved the AUC to 0.907–0.913.^184^ In an AI-integrated model combining clinical variables, radiological features, AI risk scores, and CAC findings, 10-fold cross-validation within the training cohort yielded a sensitivity of 89.53%, a specificity of 81.31%, and an AUC of 0.880; in the independent validation cohort, the model achieved a sensitivity of 82.86%, a specificity of 80.95%, and an AUC of 0.895, outperforming the Mayo model (AUC = 0.772) and the Veterans Affairs model (AUC = 0.740).^183^ These findings support diagnostic model validation, but they do not yet establish prospective outcome benefits or routine clinical utility.

Taken together, liquid biopsy and related noninvasive molecular biomarker results should be viewed as complementary biological information within CT-based screening and pulmonary nodule approaches rather than as replacements for CT or stand-alone determinants of invasive management. Their roles are assay and setting specific: plasma protein integrated classifiers have the clearest case for rule-out use in certain 8–30 mm nodules with a clinician-assessed cancer probability of ≤50%,^146,151^ whereas cfDNA methylation models combined with clinical and CT features have shown risk stratification performance in selected 5–30 mm nodule cohorts.^138,140^ cfDNA fragmentomics and plasma miRNA classifiers have been evaluated mainly as adjuncts for early detection, LDCT screening enrichment, false-positive result reduction, or risk-adapted screening intervals rather than for adjudicating CT-detected nodules or guiding the management of invasive lesions.^125,129,160,161^

EV-based assays have generated more direct nodule-oriented diagnostic evidence, including an sEV-derived long RNA signature for distinguishing T1 lung adenocarcinoma patients from those with benign pulmonary nodules in individuals with LDCT-detected indeterminate nodules^175^ and an EV membrane protein panel with a dedicated analysis for nodules ≤8 mm.^174^ CAC-based assays and integrated models have also been evaluated in cohorts with pulmonary nodules or suspected nodules, including ≤10 mm and ground-glass nodule subsets.^183–185^ Other emerging approaches remain heterogeneous: CTC detection has been explored for prospective screening,^115^ platelet RNA studies range from pancancer TEP profiling to NSCLC-focused ITGA2B models incorporating benign and small solitary nodules,^176,177^ and TCR repertoire profiling and salivary metabolomics have shown potential for risk discrimination in screening or benign lesion cohorts.^178,181^ In terms of different modalities, prospective evidence that assay-guided approaches safely reduce unnecessary PET/CT imaging, biopsies, surgeries, or excessive surveillance without delaying clinically important lung cancer remains limited.^109^

## Surgical diagnosis and intraoperative localization

When the probability of malignancy remains high after imaging-based and nonsurgical evaluation, surgical diagnosis represents the invasive end of the pulmonary nodule diagnostic pathway. Surgical diagnosis should be considered when pathological confirmation is required and percutaneous or bronchoscopic biopsy is nondiagnostic, infeasible, or inappropriate. Comprehensive preoperative assessment by experienced specialists and/or multidisciplinary teams is essential to avoid overtreatment. In certain patients, thoracoscopic diagnostic resection can provide both pathological diagnosis and definitive treatment, particularly when localization techniques facilitate the removal of small intrapulmonary nodules.^65,186–188^ However, routine surgical biopsy solely for diagnosis is discouraged when surveillance or less invasive diagnostic strategies are appropriate.^8,65^

Intraoperative ultrasound (IU) localization exploits the echogenic differences between pulmonary nodules and surrounding lung tissue and avoids radiation exposure. Previous studies have reported high localization success for ground-glass nodules (GGNs) and small pulmonary nodules, particularly after complete ipsilateral lung collapse. Although pure GGNs are often difficult to palpate, IU visualization depends on adequate lung deflation and may be technically limited by residual air, emphysema, lesion depth, or nodule composition.^186,187^ Ex vivo evaluation of a prototype ultrasound thoracoscope further supported its technical feasibility in resected human lungs, but whether this translates into clinical benefit remains to be established.^188^

## AI-assisted diagnosis and decision support

Pulmonary nodule evaluation often requires longitudinal imaging comparisons, clinical risk assessments, and structured reporting. AI tools may assist with specific steps in this pathway, including nodule detection, risk estimation, workflow triage, and emerging multimodal integration. Computer-aided detection (CADe) systems aim to detect or flag candidate nodules, whereas computer-aided diagnosis (CADx) systems seek to refine the malignancy risk of nodules that have already been detected.^189^ This distinction is clinically important. A model that increases nodule detection may reduce perceptual oversight but can also increase false-positive findings, whereas a risk stratification model would be most clinically valuable if it were prospectively shown to inform surveillance, additional imaging, biopsy, or treatment decisions without delaying cancer diagnosis.

**Fig. 3:**
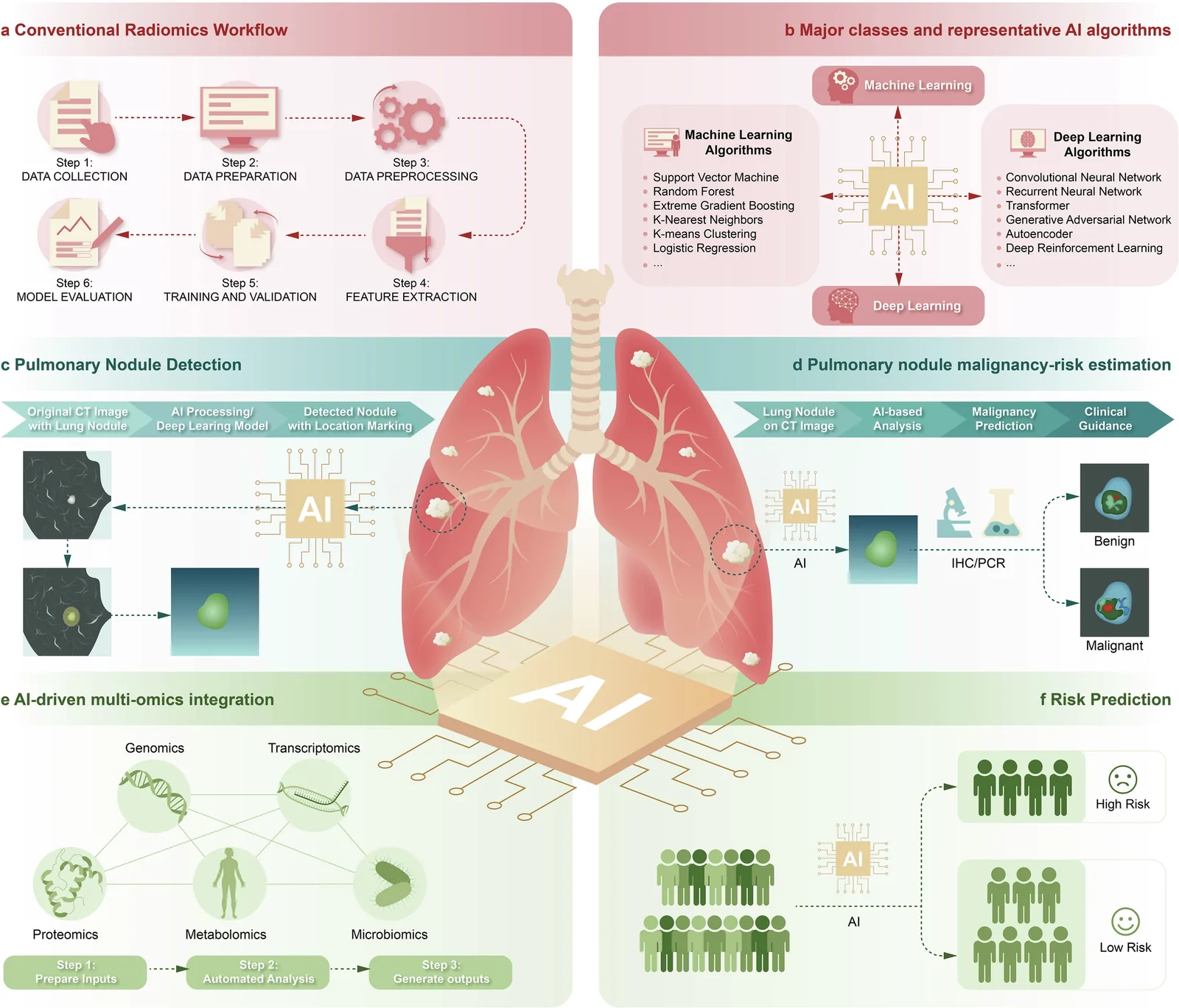
Artificial intelligence workflows and their applications to pulmonary nodule evaluation. **a** Conventional radiomic workflow comprising data collection, data preparation, image preprocessing, feature extraction, model training and validation, and performance evaluation. **b** Representative machine learning and deep learning algorithms evaluated for medical image analysis and pulmonary nodule assessment. **c** Artificial intelligence-assisted pulmonary nodule detection, localization, and segmentation on computed tomography images. **d** Artificial intelligence-based malignancy risk estimation, with subsequent pathological or molecular evaluation when clinically indicated. **e** Emerging integration of imaging with genomic, transcriptomic, proteomic, metabolomic, and microbiome data for multimodal pulmonary nodule assessment. **f** Artificial intelligence-based risk stratification to support classification into higher- and lower-risk groups. These applications should be interpreted as complementary decision support tools rather than autonomous diagnostic systems. External validation, prospective workflow assessment, calibration, cost-effectiveness analysis, and evidence of clinical utility are needed before routine implementation. AI artificial intelligence, CT computed tomography, IHC immunohistochemistry, PCR polymerase chain reaction. Created using Adobe Illustrator

For this reason, the performance of AI in pulmonary nodule management cannot be inferred from retrospective AUC, sensitivity, or specificity data alone. Recent reviews and implementation-oriented commentaries emphasize that key translational issues include external validation, robustness across scanners and populations, compatibility with established management frameworks, prospective workflow impact, and postdeployment monitoring.^190–193^ In this section, we organize AI applications into five clinically defined domains: CADe for nodule detection and workflow support, CADx for malignancy risk assessment, invasiveness and histological subtype prediction, multimodal integration of imaging and biomarkers, and practical barriers to real-world implementation. Key AI workflows and their applications in pulmonary nodule evaluation are summarized in Fig. 3.

### AI for nodule detection and workflow support (CADe)

CADe is designed primarily to detect suspicious pulmonary nodules and reduce perceptual errors in LDCT lung cancer screening and other high-volume thoracic imaging contexts. Rather than providing a definitive cancer diagnosis, it serves as a second reader aid that prompts radiologists to review potentially relevant lesions. Earlier CT-based CAD systems generally used staged pipelines comprising lung segmentation, candidate nodule detection, feature extraction, and false-positive reduction and were often based on handcrafted intensity, morphologic, and texture features combined with conventional classifiers.^194^ Some transitional systems have attempted to integrate CADe and CADx by combining nodule detection with malignancy-likelihood estimation.^195^

Deep learning has shifted this paradigm toward detection pipelines that incorporate false-positive reduction,^189,196,197^ with related AI methods also being developed for nodule segmentation, quantification, and classification.^189^ Because LDCT interpretation also requires measurement, categorization, comparison with prior scans, and management recommendations, CADe is best positioned as a reader support and workflow tool rather than a stand-alone substitute for radiologist judgment.

In different imaging contexts, available prospective and real-world studies suggest that AI assistance can improve lesion detection, although the clinical implications differ substantially by modality, population, and deployment setting. In a randomized trial of chest radiograph interpretation in a health screening population, AI assistance increased the detection of actionable pulmonary nodules compared with conventional readings without significantly increasing false referral or positive report rates.^198^ These findings support AI-assisted chest radiograph interpretation but should not be conflated with LDCT-based lung cancer screening outcomes. In a real-world Chinese physical examination CT-based lung cancer screening cohort, compared with manual interpretation, AI-based reading resulted in higher screening-positive and lung cancer detection rates, including a higher detection rate of malignant pulmonary nodules and lung cancer cases, although these findings should not be interpreted as evidence of improved long-term outcomes.^199^ Although not CADe in the strict sense, AI-enabled reconstruction may support LDCT interpretation by improving image quality, nodule conspicuity, and measurement accuracy; deep learning reconstruction has improved Lung-RADS evaluation in ultra-LDCT contexts and increased nodule detection and measurement accuracy compared with conventional reconstruction.^200,201^ On benchmark datasets, deep learning CADe systems have also achieved strong technical performance. For example, a multiview convolutional neural network reached sensitivities of 85.4% and 90.1% with one and four false-positive results per scan, respectively, on the Lung Image Database Consortium and Image Database Resource Initiative (LIDC-IDRI).^196^

Workflow-oriented studies are more directly relevant to implementation than standalone benchmark performance is. Early work explored a computer vision tool, combined with a trained technician as a first-reader strategy for lung cancer screening CT, providing a workflow-oriented precedent for computer-assisted prescreening.^202^ In 3678 baseline LDCT scans from the 4-IN-THE-LUNG-RUN trial, AI used as a first reader negative filter produced fewer negative misclassifications than radiologists did and would have resulted in fewer missed clinical referrals, suggesting a potential role in prescreening negative examinations and reducing workload.^203^ In the UKLS CT lung cancer screening study, a commercial AI system was externally evaluated against histologically proven lung cancers, suggesting a potential reduction in the workload of baseline screening interpretation.^204^ These studies support AI as a baseline rule-out or triage strategy in certain screening contexts, but they do not establish AI as an autonomous final reader across the full screening pipeline.

High nodule detection does not necessarily improve screening performance. In the Korean Lung Cancer Screening Pilot Program, a cloud-based computerized interpretation system increased the number of detected nodules and reduced interinstitutional variation in positive screening rates, but did not clearly improve Lung-RADS-based diagnostic sensitivity or specificity for lung cancer.^205^ Similarly, a Japanese external validation study revealed that a commercial deep learning nodule detector achieved high sensitivity for reference nodules and lung cancers but had a low positive predictive value and generated multiple false-positive results per CT examination.^206^ A tertiary center real-world evaluation likewise revealed that commercial nodule detection software may produce substantially higher false-positive rates than previously reported.^207^

These data define the current clinical niche of CADe: it may reduce the number of missed nodules, standardize interpretation, and support workflow efficiency, but it is not yet an autonomous screening reader. With respect to implementation, sensitivity alone is insufficient; AI systems should also be judged by their effects on workload, false-positive burden, guideline-concordant management, downstream testing, cost, and patient-relevant outcomes.^191,192^ Beyond CT-based detection, augmented reality navigation has been explored in a pilot study for intraoperative pulmonary nodule localization, although this application should be considered as downstream procedural guidance rather than for CADe, CADx, or screening interpretation.^208^

### AI for malignancy risk assessment of detected nodules (CADx)

Unlike CADe, which addresses lesion detection, CADx estimates malignancy risk after a pulmonary nodule has already been detected. Its most plausible use is in intermediate-risk nodules, where decisions about surveillance, short-interval CT, PET/CT, biopsy, or resection often vary across clinicians and guidelines. CADx should therefore be evaluated by whether it adds actionable information beyond established prediction models, such as Brock/PanCan, Mayo, and Herder, and management frameworks, such as Lung-RADS, Fleischner, and ACCP. This is especially relevant when both overinvestigation and delayed diagnosis are plausible risks.

Radiomics- and machine learning-based CADx models have shown promising discrimination, primarily in retrospective and multicohort studies. Warkentin et al. integrated radiomic and epidemiological features from four international lung cancer screening studies and reported strong malignancy prediction performance, with a test set AUC of 0.93 and similar performance for solid and SSNs.^209^ Considering small nodules, the SN-RPV model was developed for 5–15 mm solid nodules and, in retrospective decision support analyses, may assist radiologists in small nodule classification and earlier diagnostic triage.^210^ A meta-analysis of CT radiomics models reported a pooled AUC of approximately 0.91 but also identified a high risk of bias and emphasized the need for larger, prospective, and methodologically standardized validation studies.^211^ In terms of large nodules, the LIBRA study revealed that a large nodule radiomics predictive vector outperformed the median radiologist and the Brock model and suggested potential decision support value within the Herder 10–70% category despite attenuated performance in the external test set.^212^ These findings suggest that CADx is most likely to be useful not at the extremes of risk, where management is usually clearer, but in the intermediate-risk range, where additional reclassification may change clinical decisions.

Deep learning models have extended CADx from handcrafted radiomics to direct image-based risk prediction, although their incremental value remains context dependent. External validation of the lung cancer prediction convolutional neural network (LCP-CNN) showed better discrimination than the Brock model and identified a larger proportion of benign nodules as very low risk, although one false-negative cancer case occurred at the predefined rule-out threshold.^213^ In terms of indeterminate pulmonary nodules, the results of the study by Massion et al. revealed that compared with conventional risk models, LCP-CNN substantially reclassified cancers and benign nodules at guideline-relevant risk thresholds, suggesting potential but not prospectively proven management implications.^214^ A later radiological study revealed that LCP-CNN-based CAD improved clinician malignancy risk estimation for indeterminate pulmonary nodules detected by CT, increasing the average AUC from 0.82 to 0.89.^215^ In terms of LDCT screening, a deep learning model was evaluated for malignancy risk estimation of screening-detected nodules, outperforming the PanCan model in the full cohort and showing performance comparable to that of thoracic radiologists in validation subsets.^216^

End-to-end participant-level models represent a related but distinct direction and should not be interpreted as malignancy classifiers for a specified detected nodule. A three-dimensional deep learning model developed for LDCT screening predicted lung cancer risk from current and prior CT volumes and performed comparably to or better than radiologists did in certain retrospective contexts.^217^ Similarly, Sybil was developed to predict future lung cancer risk from a single LDCT examination, extending AI from visible nodule assessment toward broader imaging-based risk prediction rather than nodule-level malignancy classification.^218^ External testing of Sybil risk prediction is important because performance may vary across institutions, populations, smoking history subgroups and imaging protocols.^219^

Because pulmonary nodule management is inherently longitudinal, baseline AI-based risk prediction should be interpreted in the context of surveillance-based assessment. In a retrospective NLST-based analysis, the performance and behavior of LCP-CNN were evaluated on 10,485 nodules, supporting the role of imaging features such as nodule size and morphology in AI-based risk prediction.^220^ In the LUCINDA study, LCP-CNN achieved high discrimination for 5–15 mm nodules and, under predefined high-sensitivity thresholds, suggested that follow-up imaging might be avoided in a subset of patients.^221^ In another retrospective decision analysis of 5–15 mm SNs, threshold-based use of the LCP-CNN score suggested that follow-up CT could be reduced for low-scoring benign nodules, while high-scoring malignant nodules might undergo expedited work-up.^222^ At the participant level, DeepLR used follow-up LDCT information to predict lung cancer risk within 1–3 years and identified a large proportion of subsequent lung cancer cases, although its role is more similar to that of a screening-interval risk prediction tool than to nodule-level CADx.^223^ Prior CT information may also improve the malignancy risk estimation of pulmonary nodules detected by screening, supporting the clinical importance of the serial imaging context.^224^

C-Lung-RADS provides another example of AI moving from binary classification toward risk-adapted management. Developed from large-scale Chinese medical checkup CT data, this multistep system integrates nodule density, size, deep learning malignancy probability, clinical variables, and longitudinal growth features to stratify CT-detected pulmonary nodules.^66^ This approach is particularly relevant to Chinese health check and CT-detected nodule populations, whose risk profiles and nodule spectra may differ from those of Western smoking-defined screening cohorts. In an independent test set, C-Lung-RADS achieved higher sensitivity than Lung-RADS v2022 did (87.1% vs. 63.3%) but also had a higher false-positive rate (5.9% vs. 4.4%); therefore, its potential benefit must be interpreted alongside false-positive burden, downstream investigations, and the need for prospective clinical utility evaluation.^66^

External validation remains central to CADx translation. In three European screening cohorts, a deep learning algorithm for pulmonary nodule malignancy stratification outperformed the PanCan model for indeterminate nodules and reduced the proportion of benign nodules classified as positive while maintaining high sensitivity for cancers diagnosed within 1 year.^225^ In contrast, a multicenter gradient boosting decision tree model showed a decline from an internal test AUC of 0.873 to an external test AUC of 0.726, illustrating how performance may be attenuated across institutions and populations.^226^ Emerging approaches, such as semantically guided vision-language imaging biomarkers, may improve interpretability and cross-dataset robustness, but current evidence remains limited to early retrospective and multidataset validation rather than prospective clinical deployment.^227^

CADx is most relevant for intermediate-risk nodules, but its adoption should not be based solely on retrospective discrimination. To be clinically useful, models need calibrated risk prediction, reproducible external performance, sufficient interpretability, and prospective evidence that model-guided decisions reduce unnecessary surveillance or invasive testing without delaying the diagnosis of malignant nodules.^192,228^ The key question is whether CADx changes management safely, including de-escalation of follow-up for certain low-risk nodules, improved adherence to guideline-based care, and acceptable cost-effectiveness.^228^

### AI for predicting invasiveness and adenocarcinoma spectrum pathological categories

Beyond benign–malignant risk stratification, AI may help estimate the invasiveness of nodules across the adenocarcinoma spectrum, particularly pGGNs and PSNs. In this setting, the clinical question is not simply whether a nodule is malignant but also whether it is more likely to represent a preinvasive/minimally invasive lesion or invasive adenocarcinoma (IAC), a distinction that may inform multidisciplinary discussion of surveillance intensity, timing of intervention, and resection planning.^229^ However, such use remains investigational and requires prospective validation before guiding management decisions.^193^

Several CT-based models have shown encouraging performance for this task. For PSNs, a multicenter radiomics model combining ground-glass and solid components features has achieved an AUC of 0.98 and an accuracy of 93% for predicting IAC.^230^ For pGGNs, a multiparameter model integrating nodule size, maximum CT attenuation, and CT features outperformed models based on nodule size or maximum CT attenuation alone during external validation, with AUCs of 0.883, 0.827, and 0.791, respectively.^231^ A multicenter CT-based radiomic nomogram combined clinical–radiological variables with nodular and perinodular radiomic signatures to predict pathological invasiveness in solitary pulmonary nodules.^232^ The model achieved AUCs of 0.94, 0.90, and 0.92 in the development, internal validation, and external validation cohorts, respectively. More recently, automated quantitative analysis of pGGNs on LDCT revealed that lesion mass was the strongest predictor of invasiveness, with AUCs of 0.915, 0.913, and 0.893 in the training, testing, and external validation cohorts, respectively.^233^

Deep learning studies provide complementary evidence, mostly in retrospective cohorts enriched in surgically treated or pathologically confirmed nodules. CT-based deep learning models have been used to differentiate IAC from less invasive lesions among the SSNs considered for resection^229,234^ In subcentimeter pulmonary adenocarcinomas, 3D deep learning has also been explored to predict preoperative invasiveness.^235^

Deep learning has also been applied to finer pathological stratification, although its performance remains more limited than that of binary invasive-versus-noninvasive classification. A multicenter study evaluated ResNet-10 for three-class classification of surgically confirmed adenocarcinoma-spectrum ground-glass nodules as adenocarcinoma in situ (AIS), minimally invasive adenocarcinoma (MIA), or IAC. Compared with the radiomics and clinicoradiological machine learning models, ResNet-10 achieved a higher macro-AUC and greater accuracy.^236^ Nevertheless, its overall accuracy remained moderate.

These findings suggest that binary discrimination between invasive and noninvasive disease is currently more achievable than reliable preoperative three-class pathological classification.

Spatial context may improve CT-based assessment of invasiveness. In a single-center retrospective study of 687 GGNs, compared with a tumor-only model, a 3D DenseNet model incorporating both tumors and 5-mm peritumoral regions achieved greater discrimination for IAC versus atypical adenomatous hyperplasia (AAH)/AIS/MIA.^237^ Longitudinal reassessment remains central to screening-based nodule management: Lung-RADS v2022 emphasizes comparison with prior CT examinations, interval growth, and changes in solid components, whereas NELSON analyses support volume-based risk stratification and volume doubling time assessment for CT-detected nodules.^61,238^ Separate NELSON incidence screening data revealed that new solid nodules carry different probabilities of cancer than baseline nodules do, supporting distinct thresholds for new solid nodules.^239^

Available evidence for CT-based invasiveness prediction is still largely derived from retrospective cohorts of surgically resected or pathologically confirmed nodules. Multicenter radiomic and clinical–radiological models have been developed for PSNs, pure GGNs, and solitary pulmonary nodules.^230–232^ For LDCT screening-detected pGGNs, AI-derived quantitative measures have also been evaluated in selected persistent or progressive nodules that underwent surgical resection.^233^ More recent deep learning studies have extended this work to ternary AIS/MIA/IAC classification in surgically confirmed adenocarcinoma spectrum GGNs.^236^ Such case enrichment may inflate apparent performance relative to unselected screening, incidental detection, or conservatively managed surveillance populations. Prospective longitudinal validation is therefore needed before AI- or radiomics-based models can be used with confidence to inform the timing or extent of intervention. Future AI models should therefore incorporate serial CT-derived changes in total nodule volume, volume doubling time, and solid-component size or growth, rather than relying predominantly on static features from a single examination.

### Multimodal AI integrating imaging, clinical variables, and biomarkers

CT-based AI captures nodule morphology and longitudinal imaging behavior. However, it cannot fully capture tumor-derived molecular signals or host-response biology. The translational value of multimodal models that combine imaging features with clinical variables and circulating biomarkers for pulmonary nodule risk stratification depends not only on improved apparent discrimination but also on reproducible incremental value in independent cohorts and at clinically relevant decision thresholds.

Several studies directly developed for pulmonary nodule classification support this direction. PulmoSeek Plus integrates clinical characteristics, CT imaging features, and plasma cfDNA methylation biomarkers for 5–30 mm nodules and shows strong independent validation performance and favorable sensitivity in early-stage, small, and malignant nodules.^138^ DECIPHER-NODL combined LDCT-derived radiomic features with plasma cfDNA fragmentomics, outperforming imaging-only or cfDNA-only models and incorporating an invasiveness prediction branch with stepwise score increases from AIS to MIA and IAC.^240^ ASCEND-LUNG represents a proof-of-concept, sequential screening and postscreening pulmonary nodule management framework in which an AI-aided nodule diagnostic model integrated CT images with cfDNA methylation features.^133^ These studies suggest that multimodal integration may refine nodule risk stratification, but whether it can safely reduce unnecessary surveillance, biopsy, or surgery without delaying malignant diagnosis remains to be evaluated prospectively.

Other biomarker-based machine learning studies should be interpreted as adjacent proof-of-concept evidence rather than equivalent evidence for routine pulmonary nodule management. In LDCT screening or early detection settings, blood miRNA signatures and LDCT-miRNA risk models have shown promising performance for risk stratification in certain cohorts.^161,241^ Serum metabolic fingerprints and peripheral blood immune signatures have also been evaluated for pulmonary nodule classification or the assessment of invasiveness,^242,243^ whereas salivary metabolomics and immunosensor-derived protein marker platforms remain more closely aligned with early lung cancer detection or technical diagnostic development.^244,245^ A clinically validated cfDNA fragmentome assay has also been evaluated as a blood-based adjunct to LDCT-based lung cancer screening, supporting the broader concept that molecular signals may complement imaging-based screening results.^129,134^ However, these approaches differ in their study populations, clinical endpoints, biospecimens, assay platforms, and intended uses and therefore should not be regarded as interchangeable evidence. In particular, the cfDNA fragmentome assay evaluated by Mazzone et al. was developed to augment LDCT uptake among screening-eligible individuals rather than to determine the malignancy of an already detected pulmonary nodule; the authors explicitly stated that the test results should not influence the management or malignancy assessment of LDCT-detected nodules.^129^

Several barriers still limit clinical translation. Multimodal models require assay standardization, cross-platform reproducibility, robust handling of missing data, feasible turnaround time, laboratory infrastructure, and cost-effectiveness. Many studies also use surgically enriched, case‒control, or high-prevalence cohorts, which may overestimate the positive predictive value in real-world screening or incidental nodule populations. Blood-based approaches remain particularly challenging in screening and early-stage contexts; for example, a cfDNA methylation-based multicancer early detection assay achieved very high specificity, but its sensitivity for stage I disease remained limited, particularly when assessed across all cancer types,^246^ and ISET-based CTC detection showed low baseline sensitivity for lung cancer detection in the prospective AIR screening cohort.^115^ For now, multimodal AI should be positioned as an adjunct to CT-based assessment and guideline-based management rather than as a substitute for LDCT screening or as a stand-alone basis for pulmonary nodule management decisions.

### Challenges in clinical implementation and real-world translation

When applying AI to pulmonary nodule assessment, clinical readiness is not determined solely by the retrospective AUC. Deployment requires algorithm selection, objective local validation, integration with picture archiving and communication system (PACS)/electronic medical record (EMR)/reporting systems and, where applicable, cloud-based platforms, user-facing result display, postdeployment monitoring, and governance of model updating and drift.^192,247,248^ These steps should be treated as determinants of clinical utility rather than secondary technical details.

External validation remains insufficient for many models. Pulmonary nodule AI studies often rely on retrospective datasets, internal training–testing splits, or limited external testing; favorable internal performance may not translate into cross-center robustness.^192,247,249^ This is illustrated by a semisupervised transfer learning model for benign–malignant pulmonary nodule diagnosis, the performance of which was attenuated in an independent testing dataset.^250^ A similar reduction in external performance was reported in a multicenter malignancy risk model, reinforcing the need for external and local validation before deployment rather than relying on internal performance alone.^226^

Generalizability and measurement reproducibility across scanners, vendors, and acquisition methods remain major bottlenecks in lung nodule AI-based and quantitative CT assessments.^193,251,252^ Variability in the reconstruction kernel, slice thickness, radiation dose, spatial resolution, image noise, and Hounsfield unit calibration can compromise quantitative nodule assessment, particularly volumetry and longitudinal change evaluations.^251,252^ In screening contexts, these technical factors are directly relevant to reproducible nodule volumetry, longitudinal image comparison, and stable change assessment.^193,251,252^

Population and scenario heterogeneity must be addressed explicitly. Incidentally detected nodules and screen-detected nodules arise from different clinical contexts and are managed under different frameworks, including Fleischner, ACCP, the British Thoracic Society, and Lung-RADS.^8,9,11,253^ Therefore, thresholds or models derived from screening cohorts should be applied cautiously outside their intended clinical context. Baseline and incidental nodules also differ in their risk profiles. In the NELSON trial, volume- and volume doubling time-based management helped stratify CT-detected nodules,^238^ whereas new SNs detected during incidence screening showed distinct cancer probabilities, supporting temporally specific risk assessment.^239^

Representativeness and fairness remain related concerns. The NLST was dominated by white participants, with lower representation of black and Hispanic/Latino individuals, which may limit the generalizability of models trained primarily on such datasets.^254^ Medical imaging AI models can infer patient race from images, raising concerns that models trained on insufficiently diverse data may learn population-associated or site-associated signals that could contribute to biased performance.^255^ Future studies should report subgroup performance and include cross-population validation across different smoking profiles, ethnic groups, health systems, and geographic regions.^254,255^

Workflow integration is as important as model discrimination. The benefit of AI as a second reader depends on reader experience, interface and result display design, reader burden, and how outputs are incorporated into reporting and management decisions.^192,247,256^ In a retrospective cohort study of incidental pulmonary nodule management, an AI-enabled approach for patient identification, risk stratification, and follow-up tracking increased guideline-concordant follow-up and shortened the time to NSCLC diagnosis.^257^ However, this was a retrospective analysis rather than a prospective deployment, so workflow gains should not be interpreted as definitive evidence of improved patient outcomes.

AI may also support quality assurance by identifying missed nodules or image-reported discrepancies. In a single-center retrospective study, a deep learning system was evaluated as a second reader for the automated detection of missed pulmonary nodules on routine body CT scans, including those of the chest region.^258^ In the emergency department setting, combined AI and natural language processing have also been implemented as a quality assurance program for pulmonary nodule detection.^259^ Such tools should be embedded within accountable tracking systems rather than treated as stand-alone diagnostic solutions.

Recent evidence suggests that the near-term benefit of thoracic imaging AI is more consistently seen in terms of efficiency, measurement standardization, and workflow support than in terms of hard clinical endpoints. AI-assisted chest radiograph interpretation can shorten the reading time, particularly for examinations judged normal by AI.^260,261^ For LDCT screening, assisted nodule measurement and categorization improved Lung-RADS agreement and reduced reading time,^262^ whereas cloud-based CAD and semiautomated measurement decreased interinstitutional variability in positive screening rates.^205^

However, in a Korean national screening implementation study, CAD increased nodule detection but did not improve sensitivity or specificity for lung cancer diagnosis.^205^ Prospective studies such as DOLCE are therefore important because they are designed to test whether AI affects nodule management in real-world care rather than only improving retrospective metrics.^228^

These workflow benefits will translate into adoption only if they generate measurable clinical and health economic value. Recent health technology assessments of AI-derived software for detecting and analyzing lung nodules on CT have emphasized this uncertainty: AI assistance may improve nodule detection, measurement consistency, and reading efficiency, but direct evidence for improving clinical outcomes remains limited, and cost-effectiveness appears to be setting-dependent.^263^ Future implementation studies should therefore quantify incremental value over guideline-based management, including avoidable short-interval CT, PET/CT, biopsy, surgery, diagnostic delay, radiologist workload, downstream resource use, and patient-centered outcomes.^263^

Implementation at scale is therefore not only an algorithm performance problem but also a systems problem. Large screening and nodule management programs require standardized image acquisition, reproducible quantitative measurement, long-term comparability of prior scans, IT integration, quality assurance, and accountable follow-up tracking. Volumetric measurement and quantitative imaging can support nodule management based on volume and doubling time, but this requires standardized acquisition protocols, reproducible measurement performance, and quality control procedures.^13,251,252,264^ Registry-based surveillance can support benchmarking, quality improvement, follow-up monitoring, and postdeployment evaluation. In an analysis of >1 million individuals screened with LDCT, real-world follow-up data remained incomplete, underscoring that tracking and adherence are central issues with implementation.^265^

Incidental pulmonary nodule programs provide a complementary setting for deployment because they capture patients who are outside conventional screening eligibility and begin with an already identified lesion. Patient tracking systems and computerized registries have been associated with an earlier stage of diagnosis in incidental pulmonary nodule programs.^266^ The DELUGE study compared cancers diagnosed through screening, lung nodule programs, and neither pathway, supporting organized nodule approaches as a complement to LDCT screening.^267^

Implementation-oriented frameworks are also needed. The International Association for the Study of Lung Cancer expert recommendations on terminology and Delphi-derived quality indicators provide relevant foundations for governance, audit, and quality monitoring in screening programs.^268,269^ These are not AI validation studies, but they define the quality monitoring infrastructure into which AI tools would need to fit.

Regulation, interpretability, and standardization remain key implementation challenges for radiological AI.^247,248^ Transparent reporting of model development, validation, and intended use is also needed to support reproducibility and critical appraisal.^249^ Ethical deployment requires attention to secure data governance, bias, transparency, and accountability.^247,270^ A recent summary based on the FDA AI/machine learning-enabled device list revealed 17 AI-enabled devices for chest CT, of which four target pulmonary nodule detection and measurement and one of which targets pulmonary nodule malignancy risk prediction.^192^ Regulatory clearance should therefore not be equated with clinical maturity or prospective evidence of utility. With respect to human-in-the-loop tools, clinicians generally retain the responsibility for integrating AI outputs into imaging interpretation and management recommendations, although liability allocation among clinicians, health systems, and developers is still evolving.^271^

Patient-facing implementation also deserves caution. In a web-based randomized lung cancer screening study, human–AI discordance affected patient agreement, satisfaction, and follow-up preferences.^272^ In screening-eligible or older patients, malignancy risk estimates should be interpreted in the context of life expectancy, comorbidities, and procedural tolerance.^273^ This is particularly important because LDCT screening and GGN-like lung cancers are associated with risks of overdiagnosis and overtreatment.^274,275^ AI outputs should therefore be used as decision support inputs within shared decision-making rather than as stand-alone indicators of biopsy or treatment.^271,272^

Finally, public datasets have accelerated algorithm development, but are insufficient for establishing clinical readiness. LIDC-IDRI, LUNA16, and ANODE09 provide important foundations for benchmarking, but each has limitations in terms of sample size, representativeness, annotation consistency, and clinical context diversity.^276–278^ Therefore, performance on public benchmarks should not be viewed as a substitute for multicenter external validation, local calibration, or prospective workflow evaluation. In practice, AI outputs should still be interpreted alongside established risk assessment tools and guideline frameworks such as Lung-RADS. In a model-based baseline screening analysis, an AI-informed Lung-RADS strategy maintained sensitivity while improving specificity and reducing estimated follow-up costs, supporting the plausibility of hybrid rather than autonomous management approaches.^279^

Ultimately, real-world translation of AI for pulmonary nodule assessment should be understood as a systems problem involving algorithm performance, validation, acquisition variability, workflow integration, and clinical decision-making rather than as a direct extrapolation from retrospective benchmark performance alone.

### Future directions: from technical performance to clinical applications

AI approaches are increasingly being evaluated across several parts of the pulmonary nodule pathway, from detection and malignancy risk stratification to longitudinal risk assessment and workflow integration. The priority should shift from retrospective AUC alone toward clinically relevant decisions: reducing unnecessary CT, PET/CT, biopsy, or resection; preserving sensitivity for clinically important cancers; shortening the diagnostic delay for high-risk nodules; and improving guideline-concordant follow-up. Risk-based models have already shaped screening eligibility,^16,28,37,38^ and imaging-based AI is being explored to extend this principle toward individualized screening intervals and time-updated risk estimation.^223,280^ For incidental nodules, DOLCE is designed to evaluate an AI-assisted CT-based lung cancer prediction tool within real-world management.^228^ Workflow and guideline adherence studies underscore the need to test decision support tools within clinical workflows rather than as isolated classifiers.^281–284^

A near-term opportunity is the reclassification of intermediate-risk nodules, where management remains variable and additional information is most likely to change care. Very low- and very high-risk nodules are often adequately managed by existing thresholds, whereas intermediate-risk nodules require evidence from prospective workflows to determine whether AI-guided decisions can reduce avoidable surveillance or invasive testing without delaying cancer diagnosis.^228^ In screening pathways, time-updated prediction may also support individualized screening intervals and follow-up planning, but this application should be evaluated by interval cancer risk, delayed diagnosis, downstream testing, resource use, and patient-centered outcomes rather than by static single-scan discrimination alone.^280,285^

Validation must reflect real-world heterogeneity. Screening-detected and incidental nodules differ in their populations, pretest probabilities, and management frameworks. Heavy smokers, never-smokers, older patients with comorbidities, Asian cohorts enriched in SSNs, and hospital-based incidentaloma populations should not be treated interchangeably. Deployable tools should demonstrate calibration, subgroup performance, interpretability, and robustness across scanners, vendors, protocols, and institutions.^247,248^ Implementation should also be tested directly, including local validation and PACS/EMR integration.^192,248^ Postdeployment evaluation should address drift monitoring, registry-supported surveillance, downstream workload, false-positives, and medicolegal implications.^192,247^

Multimodal integration remains promising but should be framed as investigational. In pulmonary nodule cohorts, CT-integrated cfDNA methylation or fragmentomics models have been reported to improve the discrimination of benign and malignant nodules, but their clinical actionability requires validation at predefined thresholds.^140,240^ Proteomic and metabolomic approaches may provide additional nonimaging data for nodule risk refinement, although current evidence has not yet established their management-changing clinical utility.^145,242^ In screening-related contexts, miRNA-, protein-, and cfDNA fragmentome-based assays support the broader concept that molecular biomarkers may complement LDCT-based risk stratification, but these data should not be directly extrapolated to pulmonary nodule management.^129,150,161^

cfDNA fragmentome analysis may complement LDCT-based screening, but should not be used to characterize individual CT-detected nodules.^129^ Separately, resected GGNs/SSNs provide a research context for imaging–molecular risk stratification, with genomic and transcriptomic studies describing molecular evolution across AAH, AIS, MIA, and invasive adenocarcinoma.^286,287^ Radiogenomics studies have linked SSN and ground-glass opacity (GGO) imaging phenotypes with driver alterations and molecular features.^288,289^ Immune profiling studies suggest the progressive suppression or remodeling of antitumor immunity during early adenocarcinoma development.^290,291^ Single-cell and spatial studies have further characterized immune cell states and tumor microenvironment organization in GGN-associated or early-stage lung adenocarcinoma (LUAD).^292,293^ Additional single-cell and tumor microenvironment analyses of GGN/PSN or nonsolid versus solid lesions support microenvironmental remodeling during invasive progression.^294,295^ However, these exploratory imaging–molecular approaches require prospectively validated thresholds and outcome evidence before being used to justify earlier intervention.

Beyond nodule assessment, AI may extract noncancer biomarkers from LDCT, such as indicators of cardiovascular risk.^296^ These opportunistic applications may broaden the value of LDCT but should remain secondary unless referral pathways, clinical thresholds, and outcome benefits are established. In clinical practice, pulmonary nodule AI models should be evaluated by whether they improve guideline-concordant follow-up, reduce avoidable testing, preserve timely cancer diagnosis, and support safe de-escalation for certain low-risk nodules.

## Molecular evolution, mechanistic biomarkers, and signaling pathways in adenocarcinoma spectrum pulmonary nodules

The preceding sections focused on how pulmonary nodules are detected, characterized, and risk-stratified. A complementary question is which biological processes distinguish indolent persistent subsolid nodules from lesions that acquire invasive potential. Adenocarcinoma spectrum subsolid nodules provide a clinically relevant window into early oncogenic signaling, clonal evolution, immune escape, stromal and vascular remodeling, and adaptive survival pathways. These molecular features may inform the identification of future biomarkers and development of therapeutic approaches, although most have not been validated as stand-alone tools for pulmonary nodule management.

### Early driver events in adenocarcinoma spectrum SSNs

Among persistent SSNs, adenocarcinoma spectrum lesions may fall within the pathological continuum from AAH to AIS, MIA, or IAC.^286,297,298^ Radiologically, this process is usually reflected by an increasing solid component within ground-glass nodules or SSNs rather than a strictly linear transition in all cases, suggesting an association between radiological progression, molecular evolution, and clonal selection.^287,288,299,300^ EGFR mutation is among the most frequent early clonal events in adenocarcinoma-spectrum lesions, whereas broader alterations in the RTK/RAS axis contribute to the genomic heterogeneity of ground-glass opacity-associated pulmonary nodules.^286,288,289^ In a whole-exome sequencing study of 154 SSNs from 120 treatment-naïve patients, Li et al. identified EGFR, RBM10, TP53, STK11, and KRAS as the most frequently mutated genes, with EGFR mutations detected in ~50% of cases.^288^ These findings indicate that resected or molecularly profiled SSNs may already carry a recognizable early driver mutation. An increasing solid component ratio was associated with higher mutation frequencies of EGFR, TP53, RBM10, and ARID1B, together with an increased somatic mutation burden, suggesting that radiological “solidification” is associated with greater molecular complexity and histological invasiveness.^288,300^

Beyond point mutations, copy number alterations, structural variation changes, chromosomal instability, allelic imbalance, and mutational processes such as APOBEC-related signatures may accumulate during progression from preinvasive lesions to invasive adenocarcinoma.^287,299,301^ Allelic imbalance has been detected in premalignant AAH lesions, supporting the presence of chromosomal-level alterations before overt invasion.^301^ HLA loss of heterozygosity is a recognized mechanism of immune escape in lung cancer evolution, although direct evidence in pulmonary nodule-specific cohorts remains limited.^302^ Collectively, alterations in the expression of EGFR and other RTK/RAS axes are more closely linked to initiation, whereas alterations in TP53, cell cycle dysregulation, genomic instability, stress-response pathways, and invasion-associated transcriptional programs are more closely related to invasive transformation and high-risk phenotypes.^286,298,300^ The progression of adenocarcinoma spectrum SSNs is therefore best interpreted as a staged evolutionary process in which early oncogenic signaling is followed by increased genomic complexity, immune escape, and invasion-promoting molecular programs.^286,287,300^

### Immune microenvironment remodeling and immune evasion

Single-cell and spatial multiomics studies have indicated that GGO-associated lung adenocarcinoma is not immunologically inert; instead, altered immunosurveillance can be detected from the preinvasive or early invasive stages.^290–292^ Early lesions may retain features of adaptive immune activation, including T- and B-cell responses and lymphocyte-enriched spatial niches.^292,295,298^ With progression toward invasive LUAD, cytotoxic immune function may decline, and regulatory T-cell populations may expand,^290,291,293^ while the spatial organization of the immune microenvironment and myeloid populations is progressively remodeled.^292–294^ Tumor evolution may also involve the selection of immune evasion mechanisms, including HLA loss.^291,302^ Tertiary lymphoid structures (TLSs) may emerge during early LUAD development, with evidence that T follicular helper (Tfh)-dependent TLSs can support antitumor immunity.^292,303^

Dejima et al. reported that immune evolution from preneoplasia to invasive adenocarcinoma is characterized by a continuum of immune remodeling, with less effective and more intensively regulated antitumor immunity in later-stage disease.^291^ Spatial profiling suggested that the transition from noninvasive to invasive disease involves coordinated remodeling of tumor, immune, stromal, and vascular compartments.^292^ In GGN-associated, part-solid, and metastatic LUAD samples, Ren et al. observed stage- and phenotype-dependent immune niche transitions, including changes in CXCL9+ and TREM2+ tumor-associated macrophages and IFN-γ/STAT1-associated immune activation.^294^ The results of digital immune inference studies are consistent with this pattern: low-risk early LUAD subtypes show lower macrophage infiltration and a higher proportion of resting mast cells, whereas high-risk subtypes are more closely associated with an immunosuppressive context.^304^

Mechanistically, HOXA10 has recently been implicated in immune evasion in early LUAD. Through activation of the NF-κB/CCL2 axis, HOXA10 may promote the recruitment of immunosuppressive macrophages and reduce CD8 + T-cell infiltration and cytotoxicity.^305^ These findings suggest that immune remodeling can be detected before overt invasion and may be associated with, and potentially contribute to, pulmonary nodule progression; however, its causal role requires functional validation.^290,291,305^

### Remodeling of stroma- and vasculature-related pathways

Stromal and vascular remodeling are associated with, and may facilitate, the invasive transition of pulmonary nodules across the adenocarcinoma spectrum.^295,306,307^ Spatial and multiomic studies have shown that extracellular matrix remodeling, fibroblast activation, endothelial remodeling, and immune cell redistribution accompany the transition from preinvasive to invasive LUAD.^292,295^

Xie et al. reported an increased proportion of endothelial tip cells in early-stage LUAD and that PLVAP, which is enriched in tumor endothelial cells, can be induced by tumor-derived TGF-β1. The TGF-β1-PLVAP axis increases endothelial migration, invasion, and tube formation and may promote the invasiveness of adjacent tumor cells, supporting a potential active role for vascular remodeling in early malignant progression.^306^ Cancer-associated fibroblast (CAF)-mediated extracellular matrix reprogramming also contributes to this transition. Zhou et al. showed that SUSD2 hypersialylation, mediated in part by ST3GAL4, promotes extracellular matrix remodeling and protumorigenic CAF activation through AKT- and Smad2-related signaling, thereby supporting early LUAD progression in experimental analyses.^307^ These data link glycosylation programs, TGF-β-related signaling, endothelial plasticity, and CAF secretory states to the stromal evolution of pulmonary nodules.^295,306,307^

### Cell death escape and adaptive pathways associated with progression

TP53 alterations are associated with invasiveness and genomic instability during early LUAD evolution,^287,299,300^ and their functional consequences may extend to stress adaptation and cell death escape. In lung cancer models, Peng et al. demonstrated that TP53 mutations relieve the repression of FOXM1 and activate a mitogen-activated protein kinase-related stress-protective program through the FOXM1/MEF2C axis, increasing tolerance to ferroptosis-inducing conditions.^308^ Wild-type TP53 suppresses FOXM1 transcription by binding to PGC-1α, whereas mutant TP53 loses this regulatory capacity, leading to increased FOXM1 and MEF2C expression, reduced lipid ROS and malondialdehyde levels, and diminished sensitivity to erastin- or RSL3-induced ferroptosis.^308^

Although this mechanism is not specific to radiologically detected pulmonary nodules, it provides a plausible biological link between TP53 alterations, metabolic stress adaptation, and ferroptosis resistance during early LUAD progression. TP53 may therefore represent not only a marker of later genomic instability but also a potential molecular node connecting invasive transformation with adaptive cell death escape.^287,299,300,308^

In view of these molecular layers, the progression of adenocarcinoma spectrum SSNs involves coordinated changes in driver signaling, immune surveillance, stromal and vascular architecture, and regulation of cell death. Alterations in the EGFR and RTK/RAS axes are more closely related to initiation, TP53-associated programs to invasive transformation and stress adaptation, and pathways such as HOXA10/NF-κB/CCL2, TGF-β1/PLVAP, and ST3GAL4-SUSD2/AKT-Smad2 to the crosstalk among tumor cells, immune cells, the vasculature, and stroma.^300,305,306^ At present, these molecular features should be interpreted mainly as mechanistic and translational biomarkers rather than as validated stand-alone tools for pulmonary nodule management.

## Translational animal and phantom models: mechanistic and procedural boundaries

**Fig. 4:**
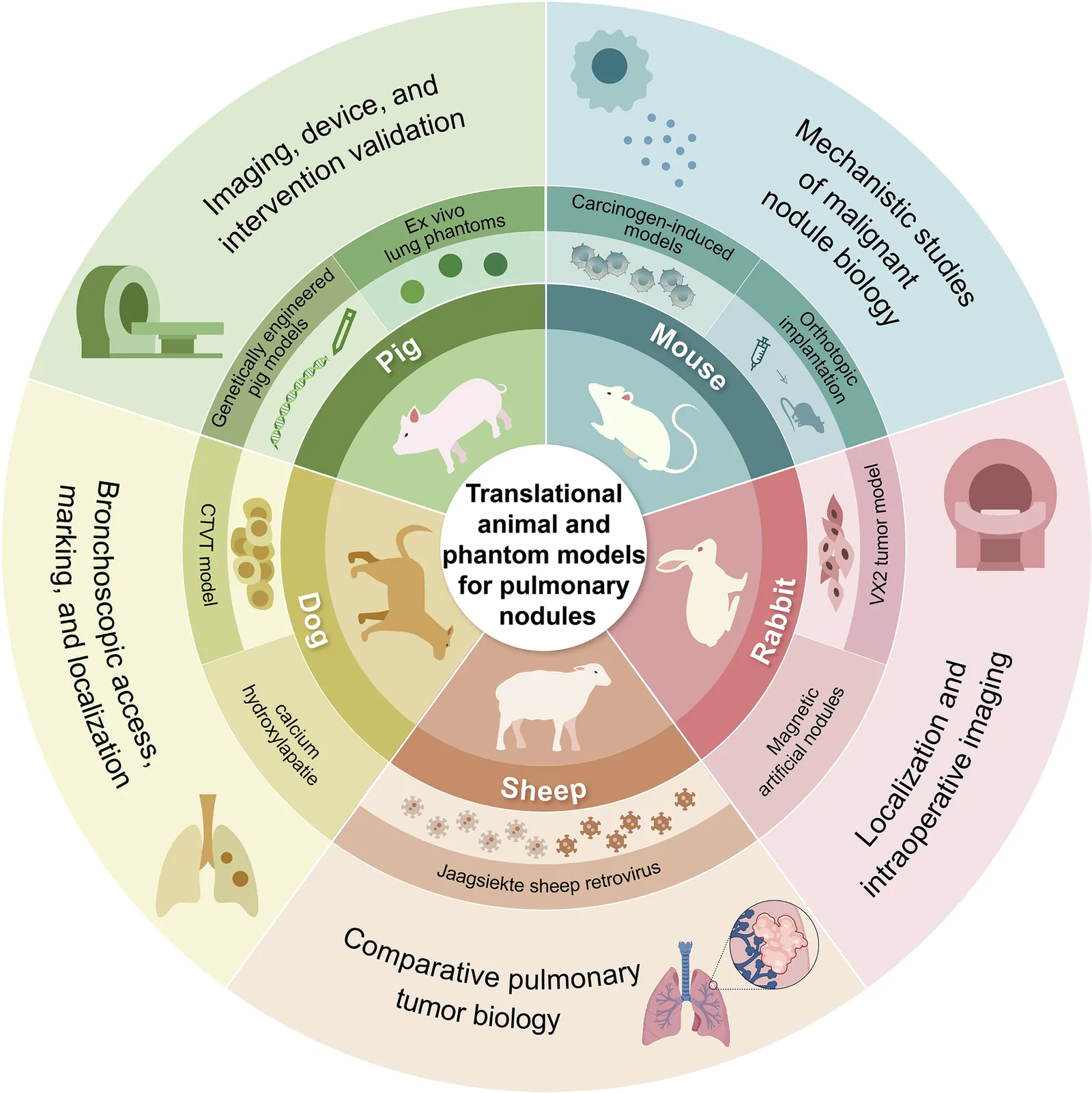
Translational animal and phantom models for pulmonary nodule research. The concentric schematic links animal species shown in the inner ring with representative animal or phantom model platforms in the middle ring and their principal translational applications in the outer ring. Carcinogen-induced mouse models support studies of malignant nodule initiation and evolution, whereas orthotopic models facilitate the evaluation of tumor growth, biomarker release, and treatment response. Genetically engineered porcine models and ex vivo lung phantoms provide anatomically relevant platforms for imaging, device, and intervention validation. Rabbit VX2 tumors and magnetic artificial nodules support localization and intraoperative imaging studies. Canine CTVT and calcium hydroxylapatite models facilitate bronchoscopic access, marking, and localization. Jaagsiekte sheep retrovirus-induced ovine pulmonary adenocarcinoma provides a naturally occurring large animal model for comparative pulmonary tumor biology. Each platform addresses a defined mechanistic or procedural question, but none fully reproduces the biological heterogeneity and longitudinal evolution of human CT-detected pulmonary nodules. CT computed tomography, CTVT canine transmissible venereal tumor, JSRV jaagsiekte sheep retrovirus. Created using Adobe Illustrator with graphical elements generated through BioRender.com

The molecular and microenvironmental processes described above are difficult to reproduce experimentally because clinically detected pulmonary nodules are imaging findings with diverse etiologies and long natural histories. Animal and phantom models therefore have value only when matched to a specific translational question, such as malignant nodule biology, biomarker development, imaging-guided localization, nonsurgical biopsy, navigation, or local intervention. These models should not be presented as direct replicas of human pulmonary nodules. Representative animal and phantom models and their translational applications are illustrated in Fig. 4, with their principal uses and limitations summarized in Table 2.

**Table 2:** Translational animal and phantom models relevant to pulmonary nodule research

Model group	Representative systems	Main use	Strengths	Limitations
Carcinogen-induced mouse models	Tg-rasH2/ENU–BHT; A/J lung tumor model	Tumor initiation, early growth and biomarker release	Immunocompetent; suitable for longitudinal micro-CT and blood sampling	Asynchronous lesions; frequent adenomas; limited similarity to human subsolid nodules
Orthotopic mouse models	Intrapulmonary implantation of lung cancer cells	Tumor growth, metastasis, treatment response	Reproducible; preserves pulmonary microenvironment	Bypasses natural initiation; human-cell models require immunodeficient hosts
Rabbit models	Magnetic artificial nodules; VX2 tumor model	Localization and intraoperative imaging	Practical for small-nodule localization studies	Limited biological fidelity to human lung nodules
Canine models	CTVT nodules; calcium hydroxylapatite or collagen pseudolesions	Bronchoscopic access, marking and localization	Human-scale airway and thoracic anatomy	Cost, ethics, immunosuppression and artificial lesions
Sheep model	JSRV-induced ovine pulmonary adenocarcinoma	Comparative lung tumor biology	Naturally occurring large-animal lung tumor model	Viral etiology; limited relevance to CT-detected indeterminate nodules
Transgenic pig models	Oncopig KRAS^G12D/TP53^R167H model	Large-animal tumor modeling and intervention testing	Human-scale anatomy; relevant driver alterations	Poor differentiation; imperfect cell-of-origin targeting; high cost
Swine and ex vivo phantom models	Pseudotumor mixtures; balloons; agar or epoxy nodules	Biopsy, navigation, sensing, ablation	Controlled lesion size, depth, and density	Lack tumor biology, immune remodeling, and biomarker shedding

Models are grouped by their main translational use. Mouse models mainly support mechanistic studies, whereas rabbit, canine, swine, and phantom models are more relevant to localization, biopsy, navigation, and intervention. No model fully reproduces the slow radiological and molecular evolution of human subsolid nodules

*BHT* butylated hydroxytoluene, *CT* computed tomography, *CTVT* canine transmissible venereal tumor, *ENU* N-ethyl-N-nitrosourea, *JSRV* jaagsiekte sheep retrovirus, *micro-CT* micro-computed tomography, *VX2* virus-induced tumor cell line 2

### Models for malignant nodule biology

Mouse models remain the primary platform for mechanistic studies because they are genetically tractable, inexpensive, and amenable to longitudinal imaging. Chemically induced models are useful for studying carcinogen-driven lung tumorigenesis in immunocompetent animals. In Tg-rasH2 mice, lung tumors can be induced by N-ethyl-N-nitrosourea and promoted by butylated hydroxytoluene.^309,310^ A/J mice administered 4-(methylnitrosamino)-1-(3-pyridyl)-1-butanone also permit serial microcomputed tomography and blood sampling during lung tumor development, supporting studies of early growth, biomarker release, and treatment response.^311^

These models inform tumor initiation and carcinogen-related biology, but their limitations are substantial. Lesions are often asynchronous and randomly distributed; many remain benign adenomas, and the induction period can be long. More importantly, they do not faithfully reproduce the slow radiological evolution of persistent subsolid nodules from atypical adenomatous hyperplasia to AIS, MIA, and invasive adenocarcinoma.

Orthotopic lung cancer models provide a complementary system in which lung cancer cells are implanted directly into the lung parenchyma.^312^ They are useful for studying local tumor growth, stromal interactions, metastasis, and therapeutic response in the pulmonary microenvironment. However, they bypass natural tumor initiation and are less suitable for modeling early nodule formation. When human tumor cells are used, immunodeficient hosts are usually needed, limiting assessments of immune surveillance and immune escape.^313–315^

### Models for imaging, localization, biopsy, and intervention

Procedural models are designed to test technologies rather than to model carcinogenesis. They are relevant to small, peripheral, deep, or impalpable nodules, for which localization, biopsy, or limited resection can be technically difficult.^316–318^

Rabbit models have been used for small nodule localization. Calcium gluconate combined with sodium alginate-Fe_3_O_4_ magnetic fluid can be used to create artificial pulmonary nodules for evaluating magnetic tracer-based localization.^319^ Rabbit VX2 tumor models have also supported the evaluation of near-infrared fluorescence guidance and thoracoscopic ultrasound localization of small peripheral lung lesions.^320,321^ These models are useful for procedural validation, but their biological relevance to naturally occurring human nodules is limited.

Canine models provide a larger airway and thoracic scale for interventional research. Pulmonary nodules can be generated by the intra-arterial or percutaneous administration of canine-transmissible venereal tumor fragments under cyclosporine-mediated immunosuppression.^322,323^ Artificial subcentimeter lesions have also been created by transbronchial implantation of calcium hydroxylapatite or CT-guided injection of colored collagen.^324,325^ These models are valuable for bronchoscopic access, localization, and marking, but are limited by immunosuppression, cost, ethical constraints, and poor fidelity to the natural early stages of lung cancer development.

Pig models are well-suited for device and intervention studies because of their thoracic size and procedural similarity to humans. The Oncopig platform enables experimental activation of Cre-inducible KRAS^G12D and TP53^R167H alterations, which are relevant to lung adenocarcinoma biology.^326,327^ Adenoviral Cre delivery through the pulmonary vasculature can induce pulmonary nodules and tumors in transgenic pigs,^328^ although the tumor morphology may be undifferentiated because of nonspecific cellular targeting and incomplete recapitulation of the relevant cells of origin.^328,329^ Nontransgenic swine and ex vivo lung phantom models, including pseudotumor mixtures, saline-filled balloons, agar gel, polymerizing epoxy nodules, and bronchoscopic or transthoracic experimental systems, have been used to evaluate biopsy, localization, navigation, sensing, and ablation techniques.^321,330–332^ These models are useful for procedural development but lack clonal evolution, immune remodeling, stromal invasion, and biomarker shedding.

Sheep infected with the jaagsiekte sheep retrovirus develop ovine pulmonary adenocarcinoma and can serve as a model for comparative studies of lung tumor biology.^333,334^ However, the viral etiology and species-specific biology limit their direct relevance to risk stratification or management of indeterminate CT-detected pulmonary nodules.

### Translational boundaries and future directions

No current animal model fully captures the clinical problem of managing pulmonary nodules: a small lesion detected by CT whose future behavior must be inferred over time. Artificial nodule models standardize procedures but have little biological content. Orthotopic and xenograft models are reproducible but bypass natural initiation. Carcinogen-induced and genetically engineered models provide mechanistic insight but do not consistently reproduce the imaging phenotype, growth kinetics, immune contexture, or histological spectrum of human subsolid nodules.

Future models should better integrate imaging and biology, ideally through immune-competent systems with longitudinal microcomputed tomography, driver-specific genetic engineering, spatial or single-cell profiling, and clinically relevant endpoints such as growth rate, emergence of a solid component, biomarker release, and response to local intervention. Until then, the results from animal studies should be interpreted as supporting evidence for mechanism, biomarker development, device testing, or procedural feasibility and not as substitutes for clinical imaging, pathology, and longitudinal cohort data.

## Management and treatment

**Fig. 5:**
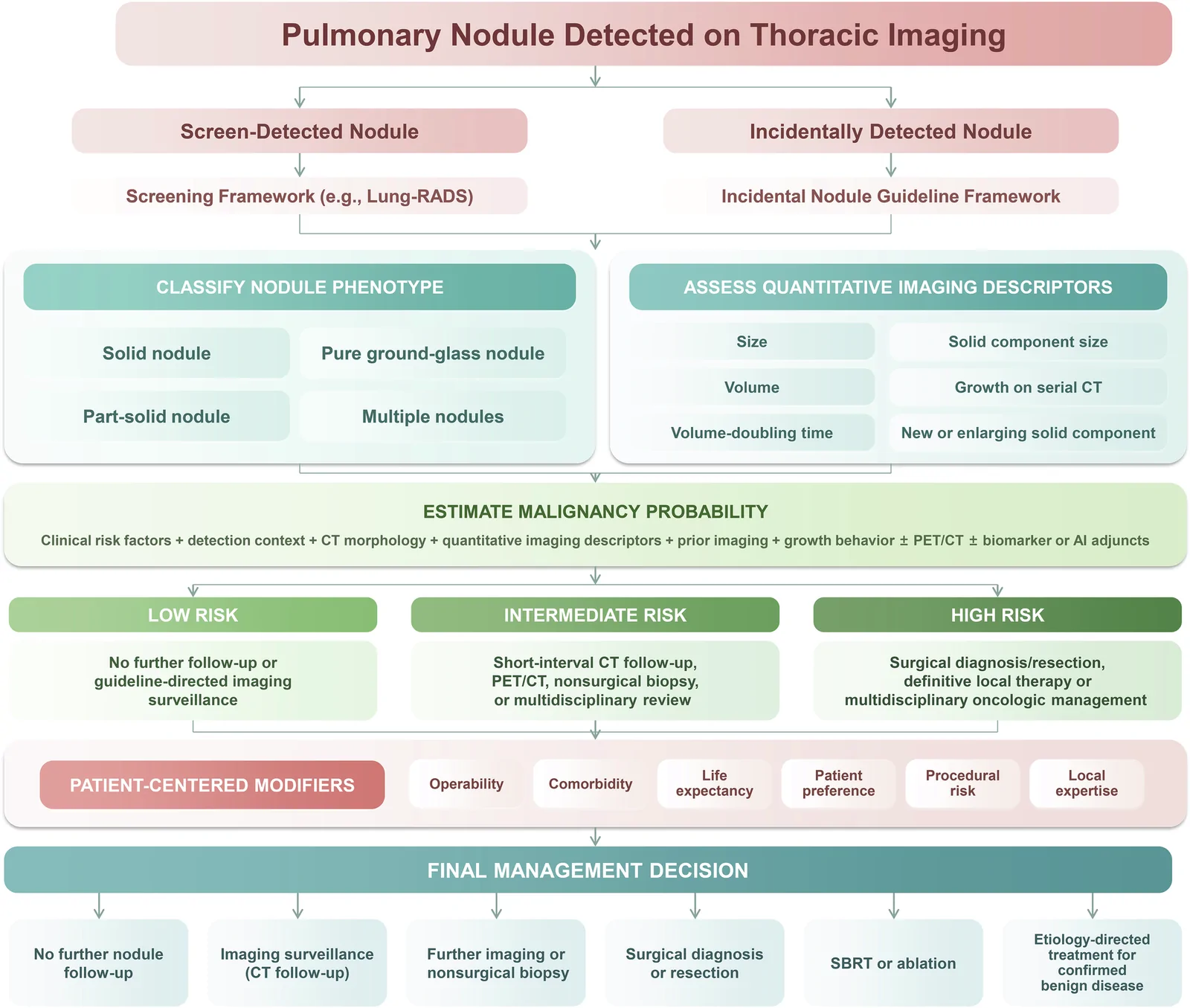
Risk-adapted framework for pulmonary nodule assessment and management. Pulmonary nodules detected on thoracic imaging are initially evaluated based on the detection context. Screen-detected nodules are generally assessed within a lung cancer screening framework such as Lung-RADS, whereas incidentally detected nodules are evaluated using an appropriate incidental nodule guideline. Initial characterization integrates nodule phenotype, size, solid component size, volume, growth on serial computed tomography, volume doubling time, and the appearance or enlargement of a solid component. The malignancy probability is then estimated by integrating clinical risk factors, detection context, computed tomography morphology, quantitative imaging descriptors, prior imaging findings, growth behavior, and certain adjuncts such as PET/CT, biomarkers, or artificial intelligence when appropriate. Management ranges from no further follow-up or imaging surveillance to additional imaging, nonsurgical biopsy, multidisciplinary review, surgical diagnosis or resection, stereotactic body radiotherapy, ablation, or etiology-directed treatment. Final decisions should account for operability, comorbidity, life expectancy, patient preference, procedural risk, and local expertise. The low-, intermediate-, and high-risk categories are conceptual and do not replace guideline-specific probability thresholds or management criteria. AI artificial intelligence, CT computed tomography, PET/CT positron emission tomography/computed tomography, SBRT stereotactic body radiotherapy. Created using Microsoft PowerPoint

Pulmonary nodule care is primarily risk-adapted management rather than treatment of all detected nodules. Individualized assessment of malignancy risk is central because it enables timely identification of clinically important early lung cancers while reducing unnecessary imaging, invasive procedures, anxiety, and the treatment of benign or indolent lesions. For nodules meeting guideline-based criteria for no further follow-up, neither surveillance nor active treatment is needed. Nodules that warrant further evaluation should undergo guideline-directed imaging surveillance or active interventions. Etiological treatment should be considered when there is clinical evidence of active infection, inflammation, or another treatable benign condition.^7,8^ For nodules with an intermediate or high suspicion of malignancy, management may include short-interval CT surveillance, PET/CT, nonsurgical biopsy, surgical diagnosis or resection, stereotactic body radiotherapy (SBRT), or ablation, depending on the estimated malignancy probability, nodule behavior, patient operability, comorbidities, life expectancy, procedural risk, local expertise, and patient preference.^8,9,85,253^ A conceptual risk-adapted approach for pulmonary nodule assessment and management is presented in Fig. 5. The NCCN and Fleischner Society recommendations are summarized in Tables 3 and 4.^8,12^ ACCP and Asian consensus recommendations are also included,^7,9^ together with the Chinese expert consensus.^65^ Table 4 also summarizes the American Association for Thoracic Surgery (AATS) recommendations for subsolid nodules.^335^

**Table 3:** Selected guideline recommendations for the management of solid pulmonary nodules

Guideline	Target population	Follow-up strategy	Intervention thresholds
NCCN	High-risk screening population	<6 mm: annual LDCT ≥6–<8 mm: follow-up LDCT in 6 months ≥8–<15 mm: follow-up LDCT in 3 months or PET/CT	≥15 mm: Contrast-enhanced CT, PET/CT, or biopsy Growing nodule >1.5 mm: LDCT in 3 months for growing solid nodules <8 mm; Contrast-enhanced CT, PET/CT, biopsy, or excision for nodules ≥8 mm
Fleischner Society	Adults with incidental nodules	<6 mm: no routine follow-up (low risk); optional CT at 12 months (high risk) 6–8 mm: CT at 6–12 months, then consider CT at 18–24 months (low risk); CT at 6–12 months, then CT at 18–24 months (high risk)	>8 mm: Consider CT at 3 months, PET/CT, or biopsy
ACCP	Individuals with pulmonary nodules	≤4 mm: no follow-up (no risk factors); CT at 12 months (with risk factors) >4–6 mm: CT at 12 months (no risk factors); CT at 6–12 months, then at 18–24 months (with risk factors) >6–8 mm: CT at 6–12 months, then at 18–24 months (no risk factors); CT at 3–6 months, then at 9–12 months and 24 months (with risk factors) >8 mm with very low probability <5%: CT at 3–6, 9–12, and 18–24 months	>8 mm with low to moderate probability 5%–65%: Functional imaging or nonsurgical biopsy >8 mm with high probability >65%: Surgical diagnosis or resection
Asian Consensus Guidelines	Asian populations	≤4 mm: consider annual CT (low risk); LDCT at 12 months, then consider annually (moderate/high risk) 4–6 mm: annual LDCT (low risk); LDCT at 6–12 and 18–24 months, then annually (moderate/high risk) 6–8 mm: LDCT at 6–12 and 18–24 months, then annually (low risk) or LDCT at 3, 6, and 12 months, then annually (moderate/high risk) >8 mm with probability < 5%: LDCT at 3–6, 9–12, 18–24 months, and then annually	>8 mm with probability 5%–60%: consider PET or nonsurgical biopsy >8 mm with probability >60%: surgical diagnosis or resection
Chinese Expert consensus	Chinese population	≤4 mm: no/optional follow-up (low risk) or annual CT (high risk) >4–6 mm: annual CT (low risk) or CT at 6–12 then at 18–24 months (high risk) 6–8 mm: CT at 6–12 months, then annually (low risk) or CT at 3–6, 9–12, then every 6 months until 2 years (high risk) 8–30 mm with very low probability < 5%: CT at 3–6, 6–9, and 9–12 months	8–30 mm with low to moderate probability 5%–65%: PET/CT or nonsurgical biopsy 8–30 mm with high probability >65%: surgical biopsy/resection

*ACCP* American College of Chest Physicians, *CT* computed tomography, *LDCT* low-dose computed tomography, *NCCN* National Comprehensive Cancer Network, *PET/CT* positron emission tomography–computed tomography

Fleischner Society recommendations shown in this table refer to single solid nodules; management of multiple solid nodules differs and is guided by the most suspicious nodule

**Table 4:** Selected guideline recommendations for the management of subsolid pulmonary nodules

Guideline	Target population	Follow-up strategy	Intervention thresholds
NCCN	High-risk screening population	• pGGNs <20 mm: annual screening LDCT; • pGGNs ≥20 mm: LDCT in 6 months; • PSNs <6 mm: annual screening LDCT; • PSNs ≥6 mm (solid component <6 mm): follow-up LDCT in 6 months; • PSNs ≥6 mm (solid component ≥6 to <8 mm): Follow-up LDCT in 3 months.	• Growing pGGNs >1.5 mm: LDCT in 6 months; consider biopsy or surgical excision for pGGNs ≥20 mm; • PSNs with solid component ≥8 mm or growing >1.5 mm: contrast-enhanced CT, PET/CT, or biopsy/resection.
Fleischner Society	Adults with incidental nodules	• pGGNs <6 mm: no routine follow-up; • pGGNs ≥6 mm: CT at 6–12 months, then every 2 years until 5 years; • PSNs <6 mm: no routine follow-up; • PSNs ≥6 mm: CT at 3–6 months to confirm persistence; if persistent and solid component <6 mm, annual CT for 5 years.	• Persistent PSNs (solid component ≥6 mm): highly suspicious; consider PET/CT, biopsy, or resection.
ACCP	Individuals with pulmonary nodules	• pGGNs ≤5 mm: no routine follow-up; • pGGNs >5 mm: annual CT for at least 3 years; • PSNs ≤8 mm: CT at 3, 12, and 24 months, then annually for 1–3 years.	• PSNs >8 mm: CT at 3 months; if persistent, evaluate with PET/CT, biopsy, or resection; • PSNs >15 mm: PET/CT, biopsy, or resection.
AATS	Individuals with subsolid nodules	• <6 mm: no routine follow-up; • Persistent pGGNs ≥6 mm: CT at 6 months, then every 12–24 months for at least 5 years; if stable, continued every 2–4 years up to 10 years; • Persistent PSNs ≥6 mm: CT at 3–6 months (shorter interval when solid part ≥6 mm or >50%), then at least annual follow-up.	• pGGNs ≥8 mm with high risk: Shortened surveillance interval, tissue sampling, or resection; • Growing pGGNs or appearance of a new solid component: tissue sampling or resection; • PSNs ≥8 mm with growth, or persistent PSNs with solid component ≥6 mm/high-risk features: Biopsy or resection.
Asian Consensus Guidelines	Asian populations	• pGGNs ≤5 mm: consider annual CT surveillance; • pGGNs >5 mm: annual CT for at least 3 years; • PSNs ≤8 mm: LDCT at 3, 12, and 24 months, then annually; • PSNs >8 mm: repeat CT at 3 months.	• Persistent PSNs >8 mm: nonsurgical biopsy or surgical resection.
Chinese Expert Consensus	Chinese population	• pGGNs ≤5 mm: CT at 6 months, then annually; • pGGNs >5–10 mm: CT at 3 months to confirm, then at 6 months and annually if stable; • PSNs ≤8 mm: CT at 3, 6, 12, and 24 months, then annually if stable; • PSNs >8 mm: CT at 3 months to rule out infection.	• pGGNs >10 mm or growing pGGNs: consider multimodal assessment, MDT, nonsurgical biopsy or surgical resection; • Persistent PSNs >8 mm, growing PSNs, or increasing/new solid component: PET-CT, nonsurgical biopsy or surgical resection.

*NCCN* National Comprehensive Cancer Network, *ACCP* American College of Chest Physicians, *AATS* American Association for Thoracic Surgery, *LDCT* low-dose computed tomography, *pGGNs* pure ground-glass nodules, *PSNs* part-solid nodules, *PET/CT* positron emission tomography–computed tomography, *CT* computed tomography

Fleischner Society recommendations shown in this table refer to single subsolid nodules; multiple subsolid nodules are managed according to the most suspicious lesion

### Management of solid nodules

Management options include imaging surveillance, nonsurgical biopsy, surgical diagnosis or resection, and local treatment modalities such as SBRT or percutaneous thermal ablation in certain patients. Additional imaging follow-up is not recommended for patients with very-low-risk nodules, such as those that have been stable for several years or have clear benign diagnostic imaging features.^85^ The management recommendations from different guidelines for solitary SNs are similar but have a few differences. The Fleischner Society recommends no follow-up for nodules <6 mm in low-risk patients,^8^ while the ACCP guidelines set this threshold at 4 mm.^9^ In contrast, the Chinese Expert Consensus recommends optional or annual follow-up for this population.^65^ For nodules <8 mm, follow-up frequency depends on risk factors. High-risk patients require shorter follow-up intervals, with specific timelines based on nodule size. For nodules >8 mm, individualized diagnostic and management strategies should be considered on the basis of malignancy probability and surgical risk. The NCCN guidelines provide recommendations for high-risk screening populations, suggesting interventions for nodules that are ≥15 mm or continue growing.^12^ Guideline-specific recommendations for solid pulmonary nodules are summarized in Table 3.

### Management of subsolid nodules

Assessment and follow-up for SSNs require thin-section (ideally 1 mm) CT scans. Multiple retrospective analyses have shown that some nodules disappeared during subsequent follow-up.^336^ These transient GGOs may be attributed to pulmonary infections and inflammatory processes.^337^ The Fleischner Society and AATS suggest that pGGNs with a diameter ≥6 mm should undergo a thin-section chest CT scan within 6–12 months or 6 months to confirm whether the nodule persists.^8,335^ If the nodule remains stable, follow-up could be performed every 12 to 24 months for at least five years. Other guidelines or consensus recommendations generally suggest annual follow-up for pGGNs with a diameter greater than 5 mm for 3 years.^7,9^ However, the NCCN guidelines recommend follow-up at 6 months for high-risk pGGNs ≥20 mm, and intervention should be considered if the pGGNs grow >1.5 mm in size.^12^ A long-term follow-up study revealed that the median time to pGGN enlargement was 71 months, with 3.9% of nodules that were stable for 10 years eventually increasing in size.^338^ pGGNs may still grow after being stable for a long time, which suggests continued follow-up. Intervention is generally considered when the nodules suddenly grow rapidly or when new solid components appear. A prospective trial confirmed that imaging follow-up could avoid unnecessary resections while ensuring survival.^339^ Randomized trials support sublobar resection for certain patients with peripheral stage IA NSCLC lesions ≤2 cm. However, these data should not be interpreted as evidence specific to subsolid nodules because trial eligibility and subgroup analyses varied by radiological phenotype, consolidation-to-tumor ratio, tumor location, and surgical margin requirements.^340,341^ In JCOG0802/WJOG4607L, segmentectomy led to a 5-year overall survival of 94.3% compared with 91.1% for lobectomy in patients with small peripheral NSCLC; these results should be discussed in the context of small peripheral NSCLC rather than as subsolid nodule-specific evidence.^340^ Moreover, complete segmentectomy or lobectomy should be considered in some cases, such as for patients with central-type SSNs >2 cm with a consolidation/tumor ratio >0.5 or for those in whom smaller resections would not achieve adequate surgical margins.^342,343^

### Management of multiple pulmonary nodules

With advances in screening technologies, the detection of multiple nodules is becoming increasingly common. In the NELSON trial, among participants with pulmonary nodules, 48.5% had two or more nodules, and 8.9% had more than four nodules.^344^ The management of multiple pulmonary nodules should involve the assessment of each lesion, with particular attention given to the dominant or most suspicious lesion. Before biopsy, resection, or combined local treatment is selected, clinicians should distinguish among synchronous primary lung cancers, intrapulmonary metastases, multifocal adenocarcinoma-spectrum lesions, and benign infectious or inflammatory nodules. When several nodules are suspicious, management should be individualized through multidisciplinary discussion, integrating radiological phenotype, growth pattern, solid component size, location, histology when available, pulmonary reserve, surgical margin feasibility, and patient preference. The primary goal is to manage high-risk lesions while preserving lung function as much as possible.^8,9^

### Definitive local treatment and adjunctive approaches

Surgery is the preferred treatment for patients with a high probability of malignancy. Notably, some patients may exceed the acceptable threshold for imaging surveillance but fall below it for surgical resection. In such cases, physicians and patients should discuss the benefits and risks of continued monitoring versus surgery. Recently, minimally invasive techniques and intraoperative navigation technologies have significantly improved the safety of surgery, but iatrogenic complications remain possible.^345–347^ Optimizing the timing of surgery is crucial, as surgical intervention that is too early may be more detrimental than beneficial. For patients who cannot tolerate surgery, SBRT or percutaneous thermal ablation may be considered. In patients with operable stage I NSCLC, a pooled analysis of data from the STARS and ROSEL phase III trials suggested that stereotactic ablative radiotherapy (SABR/SBRT) was well tolerated and associated with favorable survival, but both trials closed early due to slow accrual.^348^ The prospective MALT trial demonstrated the favorable safety and efficacy of percutaneous microwave ablation for malignant lung tumors up to 4 cm in diameter.^349^ However, SBRT and ablation techniques differ in their clinical indications; the selection criteria depend largely on nodule location, patient medical history, and diagnostic requirements. With respect to nodule location, SBRT can treat both peripheral and central lesions, albeit with a higher risk profile for central lesions. To reduce this risk, the American Society for Radiation Oncology (ASTRO) recommends avoiding a 3-fraction regimen for central lesions; instead, a 4- to 5-fraction schedule is recommended to minimize toxicity to mediastinal structures.^350^ In contrast, thermal ablation has a strong “peripheral preference” and is particularly suitable for peripheral lesions <3 cm in diameter.^351^ Notably, owing to the heat-sink effect of regional blood flow, radiofrequency ablation (RFA) is prone to incomplete ablation when the target lesion is situated near large vessels. Conversely, microwave ablation (MWA) is less susceptible to blood perfusion and is therefore more appropriate for tumors adjacent to major vessels.^352^ Furthermore, thermal ablation offers unique advantages in specific clinical scenarios, such as the need for biopsy or prior receipt of the maximum tolerated radiation dose. Lung ablation also includes cryoablation, and transbronchial ablation has been explored as an alternative route for certain peripheral lesions, particularly when avoidance of pleural puncture is desirable. However, the evidence base and technical maturity vary across modalities, and treatment selection should remain individualized based on lesion size, location, procedural feasibility, and patient fitness.^353,354^

In clinical practice in East Asia, TCM is sometimes used as supportive care during pulmonary nodule surveillance, but it has not been incorporated into standard risk-based management strategies. Because incidental pulmonary nodules are being increasingly detected with wider CT use,^355^ empirical treatment should not delay malignancy risk assessment, diagnostic clarification, guideline-directed surveillance, biopsy, surgery, or oncologic treatment when indicated. Future studies should use objective radiologic and pathologic endpoints and clearly distinguish symptom relief, psychological or quality-of-life improvement, radiologic nodule regression, and oncologic benefit. High-quality prospective randomized controlled trials with predefined radiological, pathological, oncological, and patient-reported endpoints are needed before any disease-modifying role can be established.

### Multidisciplinary care

**Fig. 6:**
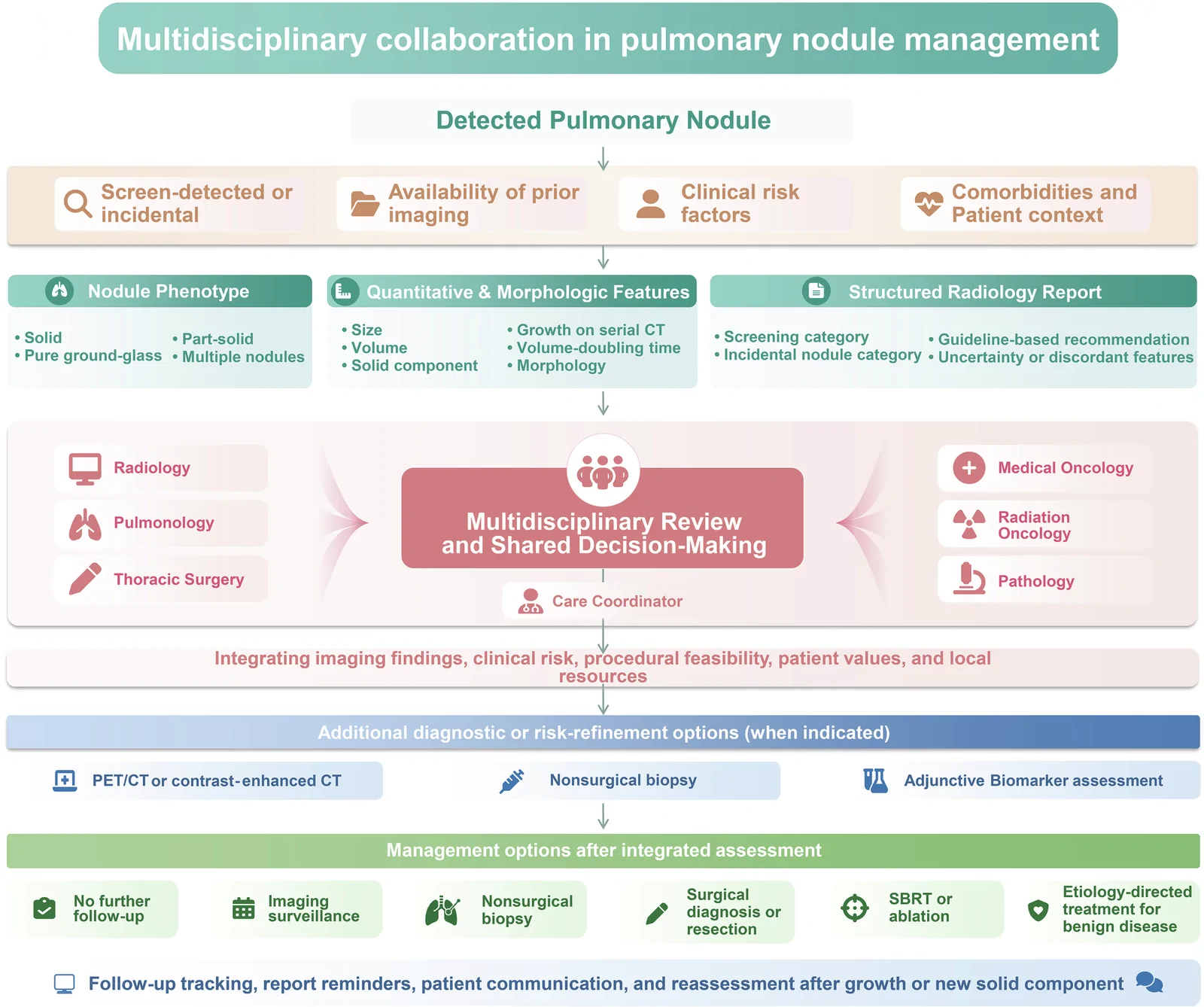
Multidisciplinary workflow for pulmonary nodule evaluation and management. After a pulmonary nodule is detected, the initial clinical context should be established, including whether the nodule was detected through screening or incidentally, the availability of prior imaging results, clinical risk factors, comorbidities, and patient-related constraints. Nodule assessment integrates the imaging phenotype, quantitative and morphological features, longitudinal changes, and structured radiological reporting, including the applicable screening or incidental nodule category, guideline-based recommendations, and areas of uncertainty. Multidisciplinary review involving radiology, pulmonology, thoracic surgery, medical oncology, radiation oncology, pathology, and care coordination supports shared decision-making by integrating estimates of malignancy probability, procedural feasibility, patient preferences, and local resources. When indicated, additional diagnostic or risk-refinement options may include PET/CT, contrast-enhanced CT, nonsurgical biopsy, and assessment of selected adjunctive biomarkers. Management may include no further follow-up, imaging surveillance, surgical diagnosis or resection, stereotactic body radiotherapy or ablation in certain nonsurgical candidates or etiology-directed treatment of confirmed benign disease. Longitudinal tracking, reminder reporting, patient communication, and reassessment after interval growth or the development of a new solid component are essential components of pulmonary nodule care. Adjunctive biomarker assessment is not intended as a stand-alone diagnostic approach. CT computed tomography, PET/CT positron emission tomography/computed tomography, SBRT stereotactic body radiotherapy. Created using Microsoft PowerPoint

Despite the availability of guideline-based algorithms, real-world care of pulmonary nodules may deviate from recommendations, resulting in both overmanagement and undermanagement.^282,283^ Furthermore, current clinical guidelines may not be suitable for some specific populations (such as patients with a history of malignancy, immunocompromised patients, or patients younger than 35 years), making it necessary to establish multidisciplinary pulmonary nodule specialty clinics. Some regions have established multidisciplinary pulmonary nodule clinics involving radiology, pulmonology, thoracic surgery, medical oncology, radiation oncology, pathology, and care coordination. These clinics integrate imaging review, malignancy risk estimation, patient fitness assessment, shared decision-making, and longitudinal tracking to support guideline-concordant management. Multidisciplinary pulmonary nodule clinics and tracking programs may improve care coordination, guideline-concordant follow-up, and the completion of recommended surveillance, although their effects on radiation exposure, downstream costs, and cancer outcomes depend on the local workflow, patient selection, and implementation quality.^282,283,356^ An integrated multidisciplinary workflow for pulmonary nodule evaluation and management is outlined in Fig. 6.

The extended growth cycle of pulmonary nodules necessitates long-term monitoring and management, posing significant challenges for patient compliance and mental health. For patients with pulmonary nodules classified according to the Fleischner Society guidelines, the consistency among clinicians, patients, and guidelines is only 46.0%.^283^ Radiologist-led follow-up communication, direct clinical decision support tools, and more structured CT reports may improve the agreement between clinical decisions and guideline recommendations.^281,284^ Shared decision-making between physicians and patients is an effective method for improving patient compliance and alleviating patients’ psychological distress.^357^ A study of 121 patients with pulmonary nodules found that 69 patients (57%) experienced psychological distress related to their condition, and 25% reported distress lasting more than two years.^358^ Effective physician‒patient communication can reduce patient distress associated with a pulmonary nodule diagnosis.^359^ Pulmonary nodule clinics, tracking systems, multidisciplinary team-based approaches, and decision support tools may improve guideline adherence and standardize follow-up.

## Summary and future perspectives

Pulmonary nodule management is shifting from size-based follow-up alone toward integrated risk assessment. Individual-level models guide selection for LDCT lung cancer screening, whereas nodule-level models and structured reporting systems guide postdetection surveillance, additional imaging, biopsy, or treatment. Noninvasive biomarkers, AI-assisted decision support, and multimodal imaging–biomarker models may add value only when they improve decisions at defined clinical steps, rather than simply increasing diagnostic discrimination in retrospective datasets.

Future work should test clinical utility rather than isolated technical performance. For AI, relevant endpoints include guideline-concordant follow-up, fewer unnecessary imaging studies or invasive procedures, timely cancer diagnosis, workflow efficiency, reader interaction, local calibration, model drift, and medicolegal accountability. For liquid biopsy and other noninvasive biomarkers, the key questions are whether they add reproducible value beyond CT-based assessments, safely reclassify intermediate-risk nodules, and reduce unnecessary biopsies or surgeries without delaying treatment of clinically important cancers. Integrated models combining imaging, clinical variables, methylation, fragmentomics, extracellular vesicle-derived analytes, protein signatures, or immune features require prospective validation at clinically relevant thresholds, including calibration, subgroup performance, cost-effectiveness, and downstream management impact.

Mechanistic and translational research remains essential for understanding why some persistent subsolid nodules remain indolent, whereas others progress toward invasive adenocarcinoma. Recent evidence implicates early oncogenic signaling, genomic instability, immune escape, stromal and vascular remodeling, and adaptive survival pathways in this transition. However, most molecular features remain translational biomarkers rather than validated stand-alone tools for clinical decision-making. Animal and phantom models can support mechanistic research, biomarker development, localization, biopsy, navigation, and local intervention, but future models should better reproduce longitudinal imaging behavior, the emergence of a solid component, immune remodeling, biomarker release, and clinically relevant intervention endpoints.

The next stage of pulmonary nodule research should focus on implementation. Effective care will require standardized image acquisition, reproducible measurement, longitudinal comparison, structured reporting, registry-supported monitoring, multidisciplinary review, and shared decision-making that incorporates malignancy probability, comorbidity, life expectancy, procedural tolerance, and patient preferences. The benchmark for new technologies should be whether they improve real-world decisions: who should undergo lung cancer screening, which nodules require closer surveillance, which patients need biopsy or treatment, and which patients can safely avoid unnecessary intervention. In this context, the goal is not simply to detect more nodules but also to improve early cancer diagnosis while reducing overdiagnosis, overtreatment, psychological distress, and avoidable harm.

## Data Availability

No new datasets were generated or analyzed in this review. All the data discussed are available from the cited publications.
